# New insights from Thailand into the maternal genetic history of Mainland Southeast Asia

**DOI:** 10.1038/s41431-018-0113-7

**Published:** 2018-02-26

**Authors:** Wibhu Kutanan, Jatupol Kampuansai, Andrea Brunelli, Silvia Ghirotto, Pittayawat Pittayaporn, Sukhum Ruangchai, Roland Schröder, Enrico Macholdt, Metawee Srikummool, Daoroong Kangwanpong, Alexander Hübner, Leonardo Arias, Mark Stoneking

**Affiliations:** 1 Department of Biology, Faculty of Science, Khon Kaen University, Khon Kaen, Thailand; 2 Department of Evolutionary Genetics, Max Planck Institute for Evolutionary Anthropology, Leipzig, Germany; 3 Department of Biology, Faculty of Science, Chiang Mai University, Chiang Mai, Thailand; 4 Center of Excellence in Bioresources for Agriculture, Industry and Medicine, Chiang Mai University, Chiang Mai, Thailand; 5 Department of Life Science and Biotechnology, University of Ferrara, Ferrara, Italy; 6 Department of Linguistics, Faculty of Arts, Chulalongkorn University, Bangkok, Thailand; 7 Material Science and Nanotechnology Program, Faculty of Science, Khon Kaen University, Khon Kaen, Thailand; 8 Department of Biochemistry, Faculty of Medical Science, Naresuan University, Phitsanulok, Thailand

## Abstract

Tai-Kadai (TK) is one of the major language families in Mainland Southeast Asia (MSEA), with a concentration in the area of Thailand and Laos. Our previous study of 1234 mtDNA genome sequences supported a demic diffusion scenario in the spread of TK languages from southern China to Laos as well as northern and northeastern Thailand. Here we add an additional 560 mtDNA genomes from 22 groups, with a focus on the TK-speaking central Thai people and the Sino-Tibetan speaking Karen. We find extensive diversity, including 62 haplogroups not reported previously from this region. Demic diffusion is still a preferable scenario for central Thais, emphasizing the expansion of TK people through MSEA, although there is also some support for gene flow between central Thai and native Austroasiatic speaking Mon and Khmer. We also tested competing models concerning the genetic relationships of groups from the major MSEA languages, and found support for an ancestral relationship of TK and Austronesian-speaking groups.

## Introduction

The geography of Thailand encompasses both upland and lowland areas, and Thailand is one of the most ethnolinguistically-diverse countries in Mainland Southeast Asia (MSEA). With a census size of ~68 million in 2015, there are 70 different recognized languages belonging to five different major language families: Tai-Kadai (TK) (90.5%), Austroasiatic (AA) (4.0%), Sino-Tibetan (ST) (3.2%), Austronesian (AN) (2.0%), and Hmong-Mien (HM) (0.3%) [1]. The majority of the people (29.72%) are called Thai or Siamese and speak a central Thai (CT) language that belongs to the TK family. Since it is the country’s official language, the number of people speaking the CT language as their primary or secondary language is ~40 million [1], or ~68% of the population.

The recorded history of the CT people or Siamese started with the Sukhothai Kingdom, around the 13th century A.D [2]. However, before the rise of the TK civilization, Thailand was under the control of Mon and Khmer people [3, 4]. Linguistic and archaeological evidence suggests that the prehistorical TK homeland was situated in the area of southeastern or southern China, and that they then spread southward to MSEA around 1–2 kya [5, 6]. This process could have occurred via demic diffusion (i.e., a migration of people from southern China, who are then the ancestors of present-day CT people), cultural diffusion (i.e., the CT ancestors were AA groups who shifted to TK languages), or continuous migration (i.e., gene flow between people from southern China and resident AA groups, so CT people have ancestry from both sources). We previously used demographic modeling to test these scenarios, using a large dataset of complete mtDNA genome sequences from Thai/Lao people, mostly from northern and northeastern Thailand, and found support for the demic diffusion model [7]. However, CT groups were not included in that study, and could have a different history.

Here we extend our previous study by adding 560 new complete mtDNA genome sequences from 22 groups (mostly from CT) speaking TK, AA, and ST languages; when combined with the previous data [7], there are a total of 1794 sequences from 73 Thai/Lao groups. We find extensive diversity in the new groups, including 62 haplogroups not found in the previous study. We use demographic modeling to test three competing scenarios (demic diffusion, cultural diffusion, and continuous migration) for the origins of CT groups. We also use demographic modeling to test competing scenarios for the genetic relationships of groups speaking languages from the major MSEA language families (TK, AA, ST, and AN) [8–11]. Our results provide new insights into the maternal genetic history of MSEA populations.

## Materials and methods

### Samples

**Fig. 1:**
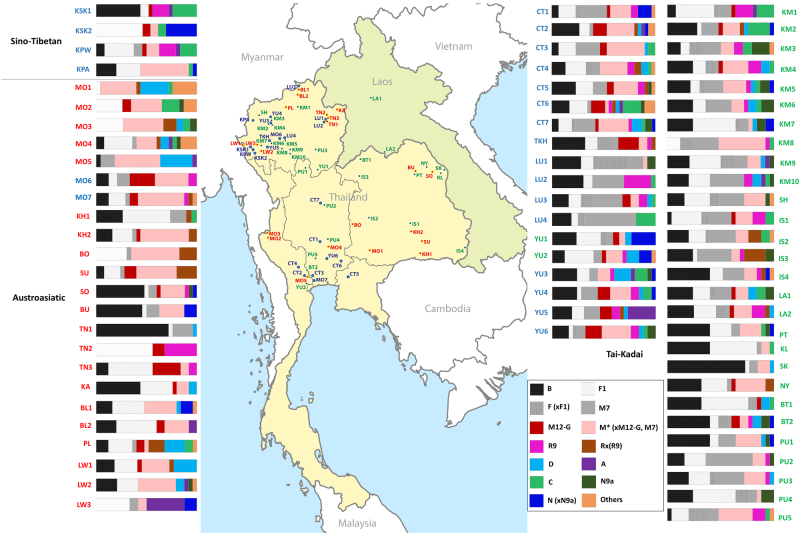
Map showing sample locations and haplogroup distributions. Blue stars indicate the 22 presently studied populations (Tai-Kadai, Austroasiatic, and Sino-Tibetan groups) while red and green circles represent Tai-Kadai and Austroasiatic populations from the previous study [7]. Population abbreviations are in Supplementary Table S1

Samples were analyzed from 560 individuals belonging to 22 populations classified into four groups: (1) the central Thais (CT) (seven populations: CT1-CT7); (2) the Mon (two populations: MO6-MO7); (3) the TK speaking groups from northern Thailand, including Yuan (four populations: YU3-YU6), Lue (four populations: LU1-LU4) and Khuen (TKH); and (4) the ST speaking Karen (four populations: KSK1, KSK2, KPW and KPA) (Table 1 and Fig. 1). Genomic DNA samples of MO6, Yuan, Lue, Khuen and Karen were from previous studies [12, 13] while the MO7 and CT groups were newly-collected saliva samples obtained with written informed consent. DNA was extracted by QIAamp DNA Midi Kit (Qiagen, Germany). This research was approved by Khon Kaen University, Chiang Mai University, Naresuan University, and the Ethics Commission of the University of Leipzig Medical Faculty.

**Table 1:** Population information and summary statistics

Population	Code	Country	Linguistic family	Linguistic branch	*N*	Haplotype information					Haplogroup information	
						Number of haplotypes	*S*	*h (*SD)	MPD (SD)	*Pi (*SD)	No of haplogroups	Haplogroup diversity (SD)
Mon	MO6	North Thailand	Austroasiatic	Monic	24	13	152	0.89 (0.05)	37.58 (16.94)	0.0023 (0.00114)	12	0.88 (0.04)
Mon	MO7	Central Thailand	Austroasiatic	Monic	25	21	271	0.99 (0.02)	39.32 (17.70)	0.0024 (0.00119)	18	0.97 (0.02)
Karen	KSK1	North Thailand	Sino-Tibetan	Karenic	25	15	123	0.91 (0.04)	30.21 (13.66)	0.0018 (0.00092)	6	0.74 (0.06)
Karen	KSK2	North Thailand	Sino-Tibetan	Karenic	13	7	99	0.83 (0.08)	31.90 (14.90)	0.0019 (0.00101)	5	0.73 (0.09)
Karen	KPW	North Thailand	Sino-Tibetan	Karenic	24	15	167	0.96 (0.02)	36.51 (16.46)	0.0022 (0.00111)	10	0.87 (0.04)
Karen	KPA	North Thailand	Sino-Tibetan	Karenic	25	21	186	0.98 (0.02)	37.03 (16.67)	0.0022 (0.00112)	12	0.92 (0.03)
Khuen	TKH	North Thailand	Tai-Kadai	Southwestern Tai	25	19	210	0.97 (0.02)	35.47 (15.98)	0.0021 (0.00108)	17	0.96 (0.02)
Lue	LU1	North Thailand	Tai-Kadai	Southwestern Tai	25	14	163	0.89 (0.05)	31.35 (14.16)	0.0019 (0.00096)	14	0.89 (0.05)
Lue	LU2	North Thailand	Tai-Kadai	Southwestern Tai	23	13	129	0.92 (0.03)	32.23 (14.59)	0.0020 (0.00099)	10	0.88 (0.04)
Lue	LU3	North Thailand	Tai-Kadai	Southwestern Tai	25	24	254	0.99 (0.01)	39.20 (17.62)	0.0024 (0.00119)	21	0.97 (0.01)
Lue	LU4	North Thailand	Tai-Kadai	Southwestern Tai	16	9	109	0.92 (0.04)	33.09 (15.24)	0.0020 (0.00103)	10	0.93 (0.04)
Yuan	YU3	North Thailand	Tai-Kadai	Southwestern Tai	25	19	236	0.97 (0.02)	34.76 (15.66)	0.0021 (0.00106)	19	0.97 (0.02)
Yuan	YU4	North Thailand	Tai-Kadai	Southwestern Tai	25	20	249	0.98 (0.02)	38.24 (17.20)	0.0023 (0.00116)	19	0.98 (0.02)
Yuan	YU5	North Thailand	Tai-Kadai	Southwestern Tai	26	20	190	0.98 (0.01)	34.80 (15.66)	0.0021 (0.00106)	15	0.93 (0.03)
Yuan	YU6	Central Thailand	Tai-Kadai	Southwestern Tai	25	14	170	0.91 (0.04)	33.23 (15.00)	0.0020 (0.00101)	13	0.90 (0.04)
Central Thai	CT1	Central Thailand	Tai-Kadai	Southwestern Tai	30	25	266	0.98 (0.02)	38.00 (17.00)	0.0023 (0.00115)	22	0.97 (0.02)
Central Thai	CT2	Central Thailand	Tai-Kadai	Southwestern Tai	30	30	346	1.00 (0.01)	38.03 (17.01)	0.0023 (0.00115)	26	0.99 (0.01)
Central Thai	CT3	Central Thailand	Tai-Kadai	Southwestern Tai	30	27	294	0.99 (0.02)	37.93 (16.96)	0.0023 (0.00114)	23	0.98 (0.01)
Central Thai	CT4	West Thailand	Tai-Kadai	Southwestern Tai	30	29	332	0.99 (0.01)	38.60 (17.26)	0.0023 (0.00116)	26	0.99 (0.01)
Central Thai	CT5	Central Thailand	Tai-Kadai	Southwestern Tai	30	28	274	0.99 (0.01)	37.16 (16.62)	0.0023 (0.00112)	22	0.98 (0.01)
Central Thai	CT6	Central Thailand	Tai-Kadai	Southwestern Tai	29	24	289	0.98 (0.02)	38.55 (17.26)	0.0023 (0.00116)	22	0.97 (0.02)
Central Thai	CT7	North Thailand	Tai-Kadai	Southwestern Tai	31	26	319	0.99 (0.01)	38.67 (17.27)	0.0023 (0.00116)	24	0.98 (0.01)

### Sequencing

We generated complete mtDNA sequences from genomic libraries with double indices and mtDNA enrichment based on protocols described previously [14, 15]. The libraries were sequenced on the Illumina Hiseq 2500. MtDNA consensus sequences were obtained as described by Arias-Alvis et al. [16]. except that Illumina standard base calling was performed using Bustard and the read length was 76 bp. Sequences were manually checked with Bioedit (www.mbio.ncsu.edu/BioEdit/bioedit.html). A multiple sequence alignment of the sequences and the Reconstructed Sapiens Reference Sequence (RSRS) [17] was obtained by MAFFT 7.271 [18]. The 560 mtDNA genomic sequences reported in this study have been deposited in NCBI GenBank under accession numbers MG272576-MG273135.

### Statistical analyses

Haplogroup assignment was performed with the online tools Haplogrep [19] and MitoTool [20]. Arlequin 3.5.1.3 was used to obtain summary statistics [21]. For the population comparisons, we included an additional 1234 mtDNA genomes from 51 Thai/Lao populations from our previous study [7] (Supplementary Table S1), for a total of 1794 sequences from 73 populations (Fig. 1). The matrix of genetic distances (*Φ*_st_, pairwise difference), permutation test, analyses of molecular variance (AMOVA), and a Mantel test of the correlation between genetic and geographic distances were also carried out with Arlequin [21]. Three types of geographic distances were computed, as previously described [7]. To get a broad picture of population relationships in Asia, we included 1936 published mtDNA genomes from 61 Asian populations (Supplementary Table S1) and calculated the *Φ*_st_ matrix by Arlequin [21].

The *Φ*_st_ distance matrix was visualized by a multi-dimensional scaling plot (MDS) using STATISTICA 10.0 (StatSoft, Inc., USA). A Discriminant Analysis of Principal Components (DAPC) was employed using the dapc function within the adegenet R package [22]. Median-joining networks [23] of haplogroups without pre-processing and post-processing steps were constructed with Network (www.fluxus-engineering.com) and visualized in Network publisher 1.3.0.0.

Bayesian skyline plots (BSP) per population and maximum clade credibility (MCC) trees per haplogroup, based on Bayesian Markov Chain Monte Carlo (MCMC) analyses, were constructed using BEAST 1.8.0 [24]. BEAST input files were created with BEAUTi v1.8.0 after first running jModel test 2.1.7 in order to choose the most suitable model of sequence evolution [25]. BSP calculations per population and the BEAST runs by haplogroup were executed with respective mutation rates of 1.708 × 10^−8^ and 9.883 × 10^−8^ for data partitioned between coding and noncoding regions [26] and Tracer 1.6 was used to generate the BSP plot from BEAST results. The Bayesian MCMC estimates (BE) and 95% highest posterior density (HPD) intervals of haplogroup coalescent times were calculated using the RSRS for rooting the tree, and the Bayesian MCC trees were assembled with TreeAnnotator and drawn with FigTree v 1.4.3.

**Fig. 2:**
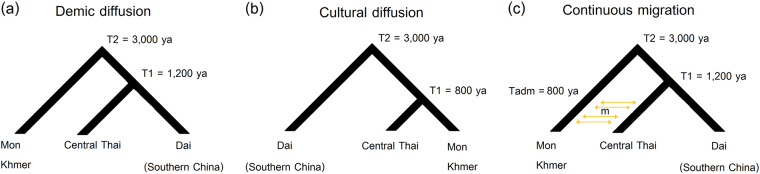
Three demographic models for the ABC analysis of CT origins: demic diffusion (**a**); cultural diffusion (**b**); and continuous migration (**c**)

**Fig. 3:**
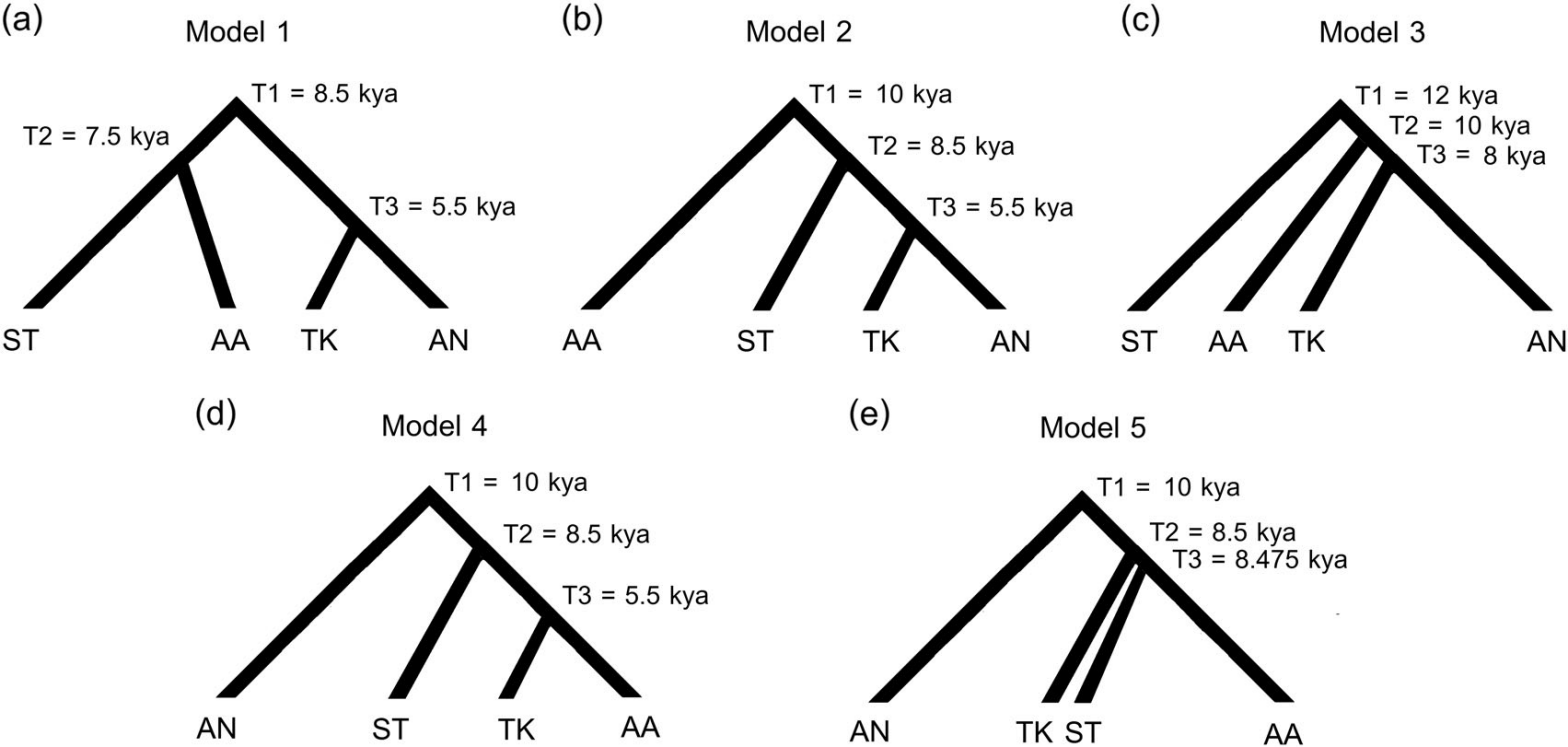
Five demographic models for the ABC analysis of the relationships of populations from four MSEA language families. Model 1 (**a**), Model 2 (**b**), and Model 3 (**c**) are based on Starosta (2005) [11], Sagart (2004, 2005) [9, 10] and Peiros (1998) [8], respectively, while Model 4 (**e**) and Model 5 (**f**) are based on the present geographic distributions of the languages (ISEA for AN and MSEA for ST, TK, and AA); see Supplementary Text for further details

An approximate Bayesian computation (ABC) approach was utilized to test different demographic scenarios concerning the origin of CT populations and the relationships between SEA language families. For the maternal origin of CT populations, we considered the same three demographic scenarios tested in our previous study for the origins of North/Northeastern Thai and Laos populations: [7] demic diffusion (Fig. 2a); an endogenous origin (with cultural diffusion of the TK language) (Fig. 2b); and continuous migration (Fig. 2c). For testing the genetic relationships of populations from the different SEA language families, we included populations speaking AA, AN, ST, and TK languages but excluded HM because of its low population size in SEA and limited mtDNA genome data. We analyzed five tree-like demographic histories based on linguistic data [8–11] for Model 1–Model 3 (Fig. 3a-c) and based on the geographic distribution of these languages for Model 4 and Model 5 (Fig. 3d,e). Since the AA, TK, and ST are the languages spoken in MSEA while AN is the major language in ISEA, Model 4 and Model 5 propose a closer affinity of AA, TK, and ST and set AN as an outgroup. Model 4 postulates an AA-TK affinity while Model 5 is a trifurcation of AA, TK and ST. Because of the computational cost of simulating a large number of complete mitochondrial sequences, we utilized a novel approach [27] based on a machine learning tool called “random forests” (RF) [28]. Additional details concerning the ABC-RF analyses are described in Supplementary Text.

## Results

### Genetic diversity and relationships

We generated 560 complete mtDNA sequences with mean coverages ranging from 54× to 3687× and identified 412 haplotypes. Genetic diversity values were lowest in the Karen group KSK2 (*h* = 0.83 ± 0.08; haplogroup diversity = 0.73 ± 0.09; *S* = 99), although this was also the group with the lowest sample size (Table 1). High genetic diversities were observed in CT populations (*h* = 1.00 ± 0.01 in CT2; haplogroup diversity = 0.99 ± 0.01 in CT2 and CT4; *S* = 346 in CT2) and Mon from central Thailand (MO7) (MPD = 39.32 ± 17.70 and *π* = 0.0024 ± 0.00119) (Table 1).

We observed 174 haplogroups among the 560 sequences (Supplementary Table S2); when combined with our previous study [7] of Thai/Lao populations, there are a total of 1794 sequences from 73 populations (Fig. 1). In total there are 1103 haplotypes and 274 haplogroups, of which 62 haplogroups were not observed in the previous study [7] (Supplementary Table S3). An analysis of haplotype sharing (Supplementary Figure S1) shows that all four Karen groups (KSK1, KSK2, KPW, and KPA) share haplotypes, indicating high gene flow among them. The Mon (MO6-MO7) shared haplotypes with several other ethnic groups, e.g., Yuan (YU) and Central Thai (CT), whereas most of the CT populations shared haplotypes more often with northeastern Thai than northern Thai groups (Supplementary Figure S1).

The AMOVA revealed that overall, 7.10% of the genetic variation is among populations (Table 2). Classifying populations by language family resulted in a slightly higher proportion of variation among groups (0.91%, *P* < 0.01) than a geographic classification (0.17%, *P* > 0.01), indicating that language family seems to be a better indicator than geography of the genetic structure of Thai/Lao populations, however there is much more variation among populations within the same group for both classifications (Table 2). Within each language family, the variation among AA groups (11.14%) was greater than that of ST (6.51%) or TK (4.59%) groups, indicating greater genetic heterogeneity of AA groups. Interestingly, we observed that the CT groups are the most homogenous of the TK groups, with only 1.64% of the variation among groups. However, Lue groups had higher heterogeneity (7.26%) than the average for TK groups (4.59%). A Mantel test for correlations between genetic and geographic distances indicates no correlation for all three types of geographic distances, i.e. great circle distance (*r* = 0.0216, *P* > 0.01), resistance distance (*r = *−0.0996, *P* > 0.01) and least-cost path distance (*r* = 0.0459, *P* > 0.01), further supporting the limited impact of geography on the genetic structure of Thai/Lao populations. Furthermore, a DAPC analysis showed that clustering groups by language family resulted in more discrimination among groups than clustering by geographic criteria (Supplementary Figure S2).

**Table 2:** AMOVA results

No. of groups	No. of groups	No. of populations	Within populations	Among populations within groups	Among groups
Total^a^	1	73	92.90	7.10*	
AA/TK/ST^a^	3	73	92.47*	6.62*	0.91*
Austroasiatic^a^	1	23	88.86	11.14*	
Mon^a^	1	7	93.10	6.90*	
H’tin^b^	1	3	74.29	25.71*	
Lawa^b^	1	3	92.22	7.78*	
Sino-Tibetan (Karen)	1	4	93.49	6.51*	
Tai-Kadai^a^	1	46	95.41	4.59*	
Lue	1	4	92.74	7.26*	
Yuan	1	6	96.10	3.90*	
Central Thai	1	7	98.36	1.64*	
Khon Mueang^b^	1	10	96.57	3.43*	
Lao Isan^b^	1	4	97.69	2.31*	
Phuan^b^	1	5	94.71	5.29*	
Geography^a^	6	73	92.85*	6.99*	0.17
Northern^a^	1	38	92.13	7.83*	
Northeastern^b^	1	16	91.29	8.71*	
Central^a^	1	14	95.84	4.16*	
Western^a^	1	3	99.12	0.88	

^*^ *P* < 0.01

^a^ Data combined from present and previous studies [7] to total 73 populations

^b^ Data set from Kutanan et al. [7]

**Fig. 4:**
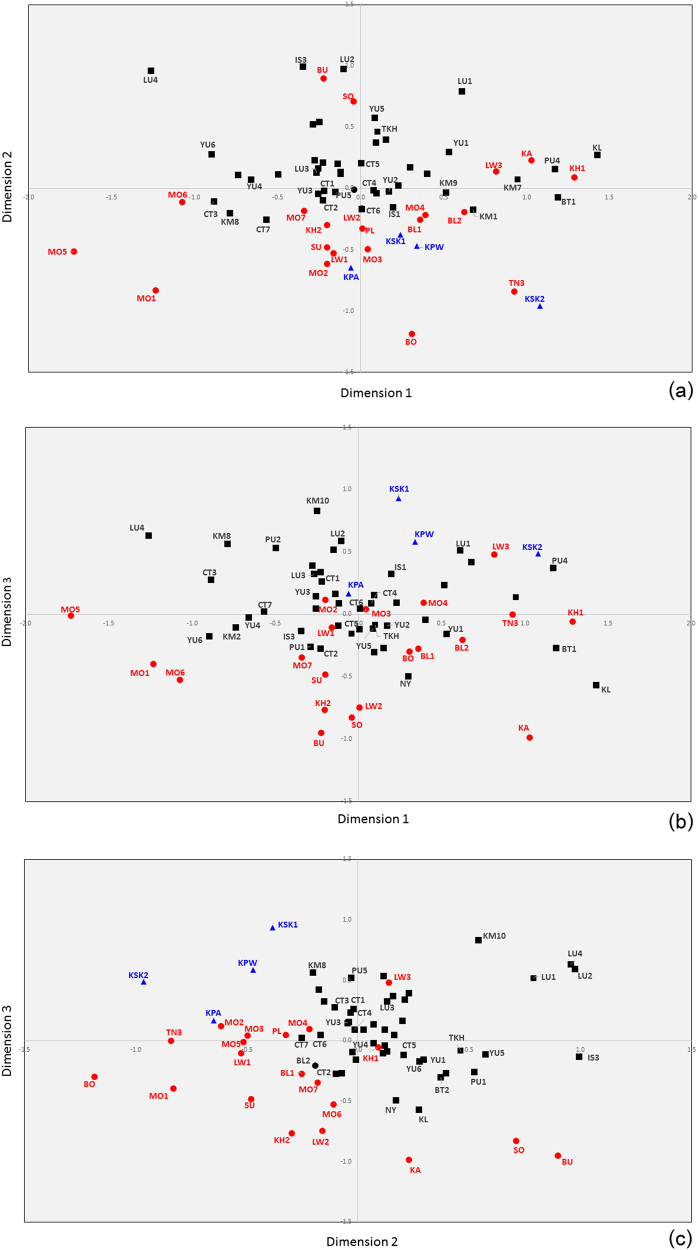
MDS plots based on the *Φ*_st_ distance matrix for 70 populations (after removal of three outliers: TN1, TN2, and SK). Red, black, and dark blue symbols indicate AA, TK, and ST populations, respectively. The stress value is 0.0804. Population abbreviations are shown in Supplementary Table S1

The MDS showed that the most differentiated groups were two H’tin groups (TN2 and TN1) and Seak (SK), as found previously [7] and in the central cloud of the plot it is difficult to see population clustering trends (Supplementary Figure S3). Genetic distance values exhibited larger significantly genetic difference of the AA populations than the TK populations, supporting greater genetic divergence of the AA groups (Supplementary Figure S4). After omitting these outlier groups (TN1, TN2, and SK), a 3-dimensional MDS provides an acceptable fit (Fig. 4a-c) and shows some clustering of populations by language family (with considerable overlap). The MDS plot of Asian populations indicated that SEA groups are separated from Indian groups; some Mon groups (MO1, MO5 and MO6) are closely related to the Indian groups as well as Myanmar (BR1 and BR2) and Cambodia (KH_C and AA_C), while the other Mon (MO2–MO4, MO7) are close to the other SEA populations (Supplementary Figure S5).

### MtDNA haplogroups

Fourteen of the 174 haplogroups occur in at least ten individuals and together account for 33.92% of the 560 sequences; these are F1a1a, B6a1a, F1f, B5a1a, F1a1a1, C7a1, C7a, M*, M12a1a, M21a, M7b1a1a3, R9b1a1a, R9b1a3, and B5a1b1 (Supplementary Table S3). These common haplogroups are mostly prevalent in AA groups (e.g., M* and M12a1a in MO6, 50.00%) and ST-speaking Karen groups (B6a1a, C7a1, R9b1a1a in KSK1, 84.00%; F1a1a in KSK2, 46.15%; F1a1a, C7a1, R9b1a1a in KPW, 70.83%; B6a1a, F1a1a1, M*, and M21a in KPA, 56.00%). These very distinct haplogroup distributions further emphasize the genetic distinctiveness of AA and ST groups.

The remaining haplogroups (66.08%), which occur in lower frequency, tend to be more widely distributed, e.g., G2a1 and basal M sublineages in MO7 and subhaplogroups F (x F1a1a and F1a1a1), M7b1a1 and B4 in Lue (LU) and Khuen (TKH) at varying frequencies (Supplementary Table S3). New subhaplogroups of B4 (B4a1a, B4a1c2, B4b1c1, B4c, B4c2c, B4g2, and B4m), F3 (F3a, F3b, F3b + 152) and M7 (M7b1a1g, M7b1a1h, M7c1c3, and M7c2b) are present mostly in TK populations (Supplementary Table S3). In agreement with the AMOVA results (Table 2), the CT groups were more similar in haplogroup distribution. The CT groups show a wide haplogroup distribution with various haplogroups occurring in a few individuals and very few haplogroups at high frequency (most are lower than 10%). Several subclades of M lineages (M12a2, M12b2, M13b1, M17c1a1, M17c1a1a, M21b2, M2a1a, M32’56, M37e2, M50a1, M51a1a, M73a1, M73b, M7, M7b, M7b1a1g, M7c1c3, and M7c2b) are newly-reported in Thai/Lao groups and are exclusively found in CT populations. Interestingly, other new haplogroups, e.g., R11’B6, R21, R23, U1a1c1a, U1a1c1d, U2a1b, and U2a2 were also observed in the CT groups (Supplementary Table S3).

In the combined Thai/Lao dataset, SEA specific haplogroups (B, F, and M7) are prevalent in almost all groups (overall frequency 55.18%), with the exception of some AA groups (i.e., Mon, Suay, Nyahkur, Khmer, and Lawa), Karen, and CT groups; these groups have other widespread haplogroups, e.g., D, M12-G, M (xM12-G, M7), A, C, and N (xN9a) (Fig. 1). Networks of common SEA specific haplogroups, e.g., B5a, F1a, F1f, and M7b, tend to exhibit star-like structures, indicative of population expansions (Supplementary Figure S6). Apart from F1a1a (xF1a1a1), other more-prevalent haplogroups of Karen (B6a1a and C7a1) do not show indications of population expansion, but rather sharing of sequences, suggesting population contraction (Supplementary Figure S6). Apart from B and F1, other lineages, that is, C7a1 and A17 and N8 which are sublineages of C, A, and N (xN9a), respectively are observed in the Karen (Fig. 1). Haplogroup C7 has a very high frequency in northeast Asia and eastern India [29] while haplogroup A was previously reported to be specific to North and Central Asia [30]. A high proportion of C and A lineages were previously observed in ST-speaking Barmar and Karen from Myanmar [31]. For the TK-specific haplogroups, i.e. B4 and M7c, there was no obvious signal of population expansion in the networks (Supplementary Figure S6).

For the combined dataset, we estimated coalescence ages of SEA haplogroups and their sublineages. We analyzed haplogroups that have additional sequences from the present study and have more than five sequences in total (Table 3). The ages of major haplogroups are generally consistent with previous studies [7]. However, we obtained more data from several sublineages which were not dated previously, e.g., B4c1b, B6a1, C4, C7a, D4a, F1c, F1e, F1g, F2, F3, F4a2, and G2a (Table 3).

**Table 3:** Coalescent ages based on Bayesian estimation with 95% highest posterior density (HPD) interval and using the 1794 Thai/Lao mtDNA sequences

Haplogroup	Sample size	Age	Lower HPD	Upper HPD
A	29	26,727.62	19,134.96	34,370.35
A17	18	14,718.80	9621.01	20,111.49
B4	111	38,117.19	30,932.00	45,747.00
B4a	24	18,917.86	12,195.35	26,000.54
B4a1c	19	14,040.58	8999.92	19,528.39
B4a1c4	17	9528.46	5673.92	13,668.78
B4b	28	24,710.85	16,441.00	33,801.00
B4b1a2a	24	15,321.55	9733.91	20,731.42
B4c	31	30,431.00	21,942.00	39,431.00
B4c2	18	12,761.47	7702.42	18,226.73
B4c1b	12	19,240.12	13,310.49	25,478.94
B4c1b2a	8	6138.65	2610.16	10,246.61
B4e	7	18,858.29	11,944.27	26,176.60
B4g	16	20,907.02	14,668.34	27,536.96
B4g1a	9	14,489.99	8675.18	20,344.45
B5	201	36,842.00	25,885.72	48,319.25
B5a	199	23,148.45	16,360.26	30,563.55
B5a1a	84	10,528.38	7009.64	14,495.93
B5a1b1	36	13,822.52	8588.07	20,104.00
B5a1d	56	11,062.58	6131.41	16,415.04
B6	63	26,393.00	17,899.18	35,489.50
B6a	62	26,070.00	17,489.66	37,976.56
B6a1	30	14,238.58	9056.86	20,278.34
B6a1a	25	7767.77	4262.58	11,673.23
C	68	25,440.22	17,812.10	33,715.36
C4	5	15,623.14	9466.73	22,086.5
C7	63	17,656.94	12,358.50	23,271.62
C7a	54	13,603.14	9194.84	18,382.15
C7a1	23	10,367.90	6654.91	14,597.69
C7a2	12	10,386.35	6153.21	14,742.98
D	74	36,798.49	27,898.26	46,589.35
D4	64	25,798.50	20,509.37	31,783.61
D4a	9	9859.99	5376.12	14,845.92
D4e	12	17,624.07	11,995.21	23,539.76
D4e1a	9	9745.70	4560.27	12,559.22
D4g2a1	9	10,492.59	6241.66	15,288.36
D4h	5	16,952.97	10,817.29	23,104.15
D4j	17	18,371.55	12,999.08	24,001.54
D4j1	13	15,823.95	10,608.52	21,007.27
D4j1a1	9	6358.27	2908.62	9832.51
D5	10	25,766.14	18,288.75	33,469.45
D5b	9	16,030.28	10,117.31	21,638.32
F1	348	32,264.31	24,186.28	41,022.47
F1a	233	17,597.91	12,944.06	23,163.01
F1a1a	173	12,638.86	8885.19	17,132.37
F1a1a1	85	10,369.11	7590.21	11,810.60
F1a1a (xF1a1a1)	88	11,109.26	7478.32	12,625.46
F1a1d	18	6483.33	2907.29	10,528.77
F1a2	9	2567.61	1266.12	4004.97
F1a3	17	10843.88	5123.02	17179.15
F1c	6	11,469.20	5757.86	17,714.81
F1e	7	19,513.31	13,131.04	26,560.48
F1f	84	10,980.60	7235.09	15,626.73
F1g	7	7927.03	3268.77	13,610.44
F2	21	23,935.18	17,170.83	31,353.49
F2b1	10	12,369.01	7203.14	17,946.33
F3	24	34,837.55	25,447.52	44,537.38
F3a	21	28,288.93	19,595.19	36,229.15
F3a1	20	19,112.58	12,812.29	25,873.93
F4a2	8	15,044.48	7932.17	23,167.29
G	29	29,188.81	21,216.46	37,267.34
G2	27	23,548.73	17,390.75	30,030.55
G2a	13	14,109.08	9224.14	19,142.22
G2a1d2	5	5799.32	2348.73	9274.34
G2a1	13	14,109.08	9224.14	19,142.22
G2b1a	11	11,690.98	6270.33	17,467.79
M*	19	54,274.26	43,577.11	66,359.72
M5	10	36,678.71	27,214.35	46,072.13
M7	212	41,391.12	31,837.71	50,939.56
M7b	171	35,034.44	26,840.83	43,472.38
M7b1a1	167	15,990.67	12,303.53	19,874.86
M7b1a1(xothers)	19	13,558.17	8123.79	19,884.27
M7b1a1(16192T)	24	12,637.55	7673.19	17,631.73
M7b1a1a3	38	12,584.53	7703.90	18,117.30
M7b1a1b	25	10,445.84	5258.37	16,254.46
M7b1a1f	18	13,245.80	7530.63	19,433.30
M7b1a1e	23	7791.66	3724.14	12,403.34
M7b1a1d1	5	2972.49	446.41	6159.96
M7c	40	30,732.28	22,122.71	39,141.31
M7c1	30	21,566.96	14,859.8	28,153.25
M7c1a	16	17,464.84	10,886.82	22,890.26
M7c1c	10	10,618.92	5486.60	16,461.94
M7c2	10	8857.81	5156.31	13,208.00
M8a2a1	12	12,289.16	6303.89	19,070.80
M9	13	25,048.34	16,817.63	33,645.48
M12-G	77	49,208.31	38,581.81	60,249.67
M12	48	34,273.83	27,438.97	41,570.70
M12a	35	31,049.21	24,795.78	37,838.12
M12a1a	26	21,687.96	16,394.10	27,437.19
M12a1b	5	21,169.00	15,368.51	27,771.39
M12b1b	8	7482.85	3500.09	11,993.80
M12b	13	25,046.13	18,605.47	31,841.06
M13	6	50,710.00	37,118.08	64,142.80
M17	18	40,904.24	30,197.33	52,184.59
M17a	5	20,009.10	13,015.45	27,964.05
M17c	13	32,177.80	23,403.54	41,810.14
M17c1a	6	17,915.50	12,186.65	24,567.08
M20	30	12,477.81	7287.09	17,537.99
M21	20	42,734.21	33,264.99	53,871.33
M21a	7	3930.28	745.70	8746.90
M21b	13	34,539.86	27,357.67	42,665.79
M24	23	19,997.93	12,330.76	28,223.99
M24a	13	9808.57	4590.87	15,938.40
M24b	10	10,410.36	5535.44	15,467.68
M51	13	29,132.45	20,474.60	38,980.81
M51a	11	23,652.72	15,649.38	30,973.61
M61	9	12,811.00	5846.11	20,533.93
M71	31	31,226.61	23,598.07	39,142.13
M71(151T)	14	23,561.12	17,922.81	29,228.41
M71a	12	23,996.16	17,978.14	29,850.89
M71a2	7	15,377.76	9811.96	21,043.64
M72	10	15,399.31	8120.81	22,767.31
M73	9	36,206.88	24,769.66	47,741.06
M74	35	34,052.07	25,392.91	42,794.43
M74a	6	9157.03	3700.32	14,608.82
M74b	26	24,068.66	18,199.97	30,801.78
M76	12	30,665.07	20,459.90	42,014.36
M91	11	35,980.00	24,612.34	48,440.13
M91a	10	15,874.00	9310.13	23,117.39
N8	8	5670.00	1800.20	10,274.52
N9a	40	23,307.91	16,466.89	31,217.48
N9a6	9	12,157.84	7080.02	17,014.10
N9a10	19	15,864.76	11,161.95	20,533.24
N10	12	51,144.71	35,516.27	65,932.28
N10a	11	11,002.31	6044.77	16,435.19
N21	15	11,924.14	7327.79	17,377.08
R9	75	36,737.77	28,196.01	45,770.54
R9b	68	32,837.96	25,372.79	40,740.86
R9b1	48	20,294.50	15,024.28	26,305.99
R9b1a	42	14,387.62	9257.45	20,045.40
R9b1a1a	12	7547.06	4217.30	11,157.00
R9b1a3	26	9062.02	5398.62	13,213.44
R9b2	18	8945.97	5003.56	13,337.12
R9c1	7	22,854.33	15,036.92	30,754.85
R22	26	39,111.69	30,325.41	49,812.23
U	8	52,604.10	41,647.27	63,469.01
W	8	13,994.04	7354.74	21,364.09

There are many lineages with ages older than 30 kya found in our Thai/Lao samples, e.g. B4, B5, D, F1, F3, M7, M*, M12, M13, M17, M21, M71, M73, M74, M91, R9, R22, N10, and U (Table 3). Many of them are major lineages and distributed in our Thai/Lao samples as well as in other SEA populations, and have been previously discussed [7]. Here, we focused on some uncommon ancient lineages, i.e., M*, M17, M21, M71, M73, M91 and U; these are described in Supplementary Text. Overall, the CT groups contrast with other Thai/Lao groups in exhibiting several ancient haplogroups (in particular basal M lineages, i.e., M13, M17, M21b, M71, M73, M91a, and U) at low frequency. Notably, M17, M21, M71, and M73 are ancient maternal lineages of SEA found in both MSEA and ISEA, reflecting linkages between the early lineages in SEA [32].

Finally, several haplogroups associated with the AS expansion from Taiwan [33–36], namely B4a1a1a, M7b3, M7c3c, E1a1a, and Y2 were not observed, suggesting that this expansion had at most a limited impact on mtDNA lineages in MSEA.

### Bayesian skyline plots

**Fig. 5:**
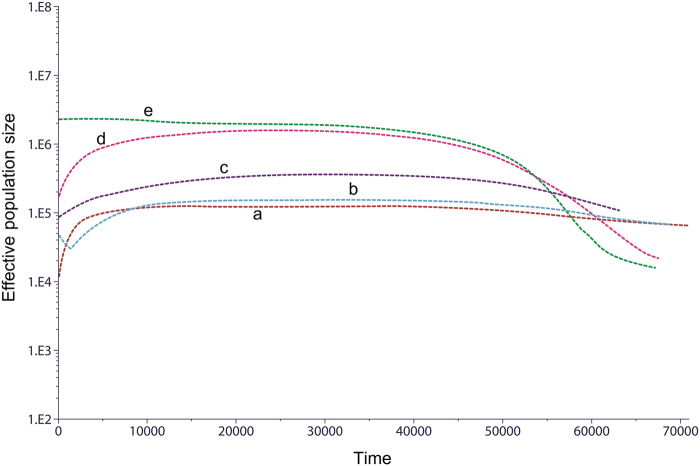
The BSP plots for five different trends found in 22 populations; KSK2, MO6, LU4 (**a**), KSK1 (**b**), KPA (**c**), KPW, MO7, KPW, TKH, LU1-LU2, YU3-YU6, CT6-CT7 (**d**), and LU3, CT1-CT5 (**e**). Population abbreviations are listed in Table 1. Each line is the median estimated maternal effective population size (y-axis) through time from the present in years (x-axis)

BSP of population size change over time were constructed for each group, and five typical patterns were observed (Fig. 5). The four Karen populations all showed different patterns: KSK2 (and also MO6 and LU4) displayed unchanged population size until ~1–2 kya followed by sharp reductions (Fig. 5, pattern a); KSK1 was also constant in size, with a sudden increase in the last 1–2 kya (Fig. 5, pattern b); KPA was basically constant in size over time (Fig. 5, pattern c); and KPW exhibited the most common pattern (also observed in MO7, KPW, TKH, LU1-LU2, YU3-YU6, CT6-CT7), consisting of population expansion between 50–60 kya, followed by a decrease in the last 5 kya (Fig. 5, pattern d). While recent reductions in population size could reflect recent bottlenecks, such changes during recent times should be interpreted cautiously as they may reflect a bias in sampling [37]. Finally, population growth without further change was found for LU3 and CT1-CT5 (Fig. 5, pattern e). The BSP plots for each individual population are depicted in Supplementary Figure S7.

### Demographic models for the origin of central Thai people

In our previous study we used demographic modeling to show that northern and northeastern Thai groups most likely originated via demic diffusion from southern China [7]. Here we use the same approach to test three demographic scenarios concerning the origins of central Thai groups: (1) descent from the prehistorical Tai stock of southern China via demic diffusion, like their neighbors in the North and Northeast of Thailand (Fig. 2a); (2) local AA groups (Mon and Khmer) who changed their identity and language via cultural diffusion to become TK groups (Fig. 2b); or (3) descent from a migration from southern China that received gene-flow from the local Mon and Khmer people (Fig. 2c). The estimated values of ABC-RF prior error rate with respect to the number of trees in the forest indicated that 500 trees were sufficient in our analyses (Supplementary Figure S8). Linear Discriminate Analysis (LDA) plot shows that the observed data fall within the distributions simulated under the three models (Supplementary Figure S9) while the confusion matrix indicates some problems in distinguishing the continuous migration model from demic diffusion (Supplementary Table S4). This result is understandable, as the two scenarios only differ for the events of migration hypothesized between the AA and CT groups. However, the demic diffusion model had the highest posterior probability at 0.604 and was selected slightly more often among the classification trees (0.515) than the continuous migration model (0.404); both of them were selected much more often than the cultural diffusion model (0.081) (Supplementary Table S5). Moreover, the estimated parameters for the continuous migration model indicate very low level of migration between CT and AA groups (Supplementary Table S6, Supplementary Figure S10). We conclude that demic diffusion, possibly with a very low level of gene flow between CT and AA groups, is the most likely scenario for the origins of central Thai populations.

### Genetic relationships of populations from different language families

We also used the demographic modeling approach to test different models for the genetic relationships of populations belonging to the four main SEA language families (TK, AA, AN, and ST). In doing so, it is important to keep in mind that we are not testing the relationships of these language families, as that would require linguistic data. However, determining the best-fitting model based on genetic relationships may help discriminate among hypotheses concerning the language family relationships that make predictions about the genetic relationships of populations speaking those languages. We tested five models of the language family relationships (Fig. 3). The observed data fall within the range of the simulated data in the LDA plot (Supplementary Figure S9) while the confusion matrix confirms our ability to distinguish amongst the different scenarios (Supplementary Table S7). The model that best fit the mtDNA genome data was Model 1, according to Starosta [11] (Fig. 3a). The posterior probability of this model is 0.657, and it was selected slightly more often among the classification trees (0.509) than Model 2 (0.311); the other models were much less often selected among the classification trees (0.037 for Model 3; 0.112 for Model 4; 0.031 for Model 5) (Supplementary Table S8). Because of the high selection frequency of Model 1 and Model 2, which have in common an ancestral relationship of TK and AN groups (Fig. 3a,b), we conclude that the TK and AN groups are descended from a common ancestral population.

## Discussion

The present study adds to our previous study of Thai/Lao mtDNA genome sequences by including 22 additional groups from Thailand, including the AA-speaking Mon (MO), ST-speaking Karen, and several TK speaking groups, especially the CT. Similar to our previous mtDNA study [7], genetic heterogeneity among populations belonging to the same ethnic groups was still observed, especially for the Karen (the hill tribes) (Figs. 1, 4 and 5). Geographic isolation and matrilocal residence appear to be important factors influencing the genetic landscape of the highlanders. For the remaining lowland groups, i.e., Mon and other TK-speaking groups, gene flow with other groups is a rather more important factor.

The Mon, who were a previously dominant group in MSEA located in present-day southern Myanmar and central Thailand since the 6 to 7th century A.D. [38], have been reported to link with Indian populations with some haplogroups, i.e., W3a1b [7]. With data from two additional Mon groups, there is still support for a connection between India and the Mon in the distribution of M subhaplogroups characteristic of South Asia or the Near East [39–41], e.g. M6a1a, M30, M40a1, M45a, and I1b (Supplementary Table S3). Genetic relationship analysis also reveals some Mon populations (MO1, MO5, MO6) clustering with Indian groups, although the other Mon groups were closely related to Thai/Lao populations (Supplementary Figure S5), possibly reflecting gene flow. Thus, based on the many older mtDNA lineages observed, the modern Mon from both Thailand and Myanmar could be an important group for further studies to reconstruct early SEA genetic history.

The Karen in Thailand are refugees who migrated from Myanmar starting from the 18th century A.D. due to the influence of Burmese [42]. However, the ancestors of the Karen probably migrated from some unknown location to Myanmar, as the Karen languages are thought to have originated somewhere in north Asia or in the Yellow River valley in China, i.e., the homeland of ST languages [43]. In agreement with previous studies of different Karen subgroups and/or different genetic markers [31, 44, 45], we find both northeast and southeast Asian components in the maternal ancestry of the Karen.

The present results emphasize the common maternal ancestry of CT and other TK speaking groups in MSEA, e.g., Laos and Southern China. Demic diffusion is still the most probable scenario for TK-speaking populations (Fig. 2a, Supplementary Table S5), possibly accompanied by some low level of gene flow with autochthonous Mon and Khmer groups. It seems that the prehistoric TK groups migrated from a homeland in south/southeast China to the area of present-day Thailand and Laos, and then split to occupy different regions of Thailand, expanding and developing their own history. During the migration and settlement period, genetic contact with the local AA people was certainly limited, but nonetheless the modeling results, haplogroup profiles and genetic diversity values all suggest some degree of admixture in the CT groups (Supplementary Table S3, Supplementary Table S6, Table 1). However, historical records indicate that large proportions of the CT groups were taken to neighboring kingdoms as war captives in multiple episodes from 500 to 300 ya [4]. The present-day CT people are probably not solely descended from the prehistorical TK groups, and admixture with local groups might have occurred starting from this time. However, in sum, cultural diffusion did not play a major role in the spread of TK languages in SEA.

Finally, we used simulations to test hypotheses concerning the genetic relationships of groups belonging to different language families. We found that Starosta’s model [11] provided the best fit to the mtDNA data; however, Sagart’s model [9, 10] was also highly supported. These two models both postulate a close linguistic affinity between TK and AN. Although genetic relatedness between TK and AN groups has been previously studied [7, 46, 47], to our knowledge this is the first study to use demographic simulations to select the best-fitting model. Our results support the genetic relatedness of TK and AN groups, which might reflect a postulated shared ancestry among the proto-Austronesian populations of coastal East Asia [48].

Specifically, the best-fitting model suggests that after separation of the prehistoric TK from AN stocks around 5–6 kya in Southeast China, the TK spread southward throughout MSEA around 1–2 kya by a demic diffusion process, accompanied by population growth but with at most minor admixture with the autochthonous AA groups. Meanwhile, the prehistorical AN ancestors entered Taiwan and dispersed southward throughout ISEA, with these two expansions later meeting in western ISEA. The lack of mtDNA haplogroups associated with the expansion out of Taiwan in our Thai/Lao samples has two possible explanations: either the Out of Taiwan expansion did not reach MSEA (at least, in the area of present-day Thailand and Laos); or, if the prehistoric AN migrated through this area, their mtDNA lineages do not survive in modern Thai/Lao populations. Ancient DNA studies in MSEA would further clarify this issue. Moreover, although mtDNA analyses are informative in elucidating genetic perspectives in geographically and linguistically related populations, they have an obvious limitation in that they only provide insights into the maternal history of populations. Future studies of Y chromosomal and genome-wide data will provide further insights into the genetic history of Thai/Lao populations and the role of factors such as post-marital residence patterns and migration in shaping the genetic structure of the region.

## Electronic supplementary material

Supplementary Information

Supplementary Table S2
