# Supplementary material for: New insights from Thailand into the maternal genetic history of Mainland Southeast Asia
Source: Eur J Hum Genet. 2018 Feb 26;26(6):898–911. doi: 10.1038/s41431-018-0113-7 (PMC5974021; doi:10.1038/s41431-018-0113-7)
Supplement: Supplementary file 1 — Supplementary Information [file 41431_2018_113_MOESM1_ESM.docx]

**Supplementary Information**

**New insights from Thailand into the maternal genetic history of Mainland Southeast Asia**

The supplementary information consists of Supplementary Text, ten Supplementary Figures and eight Supplementary Tables:

**Supplementary Text** Description of Approximate Bayesian Computation (ABC) analyses and additional details on mtDNA haplogroups.

**Supplementary Figure S1** Relative frequency of shared haplotypes within and among 73 Thai/Lao populations. Population abbreviations are in Supplementary Table S1.

**Supplementary Figure S2** Discriminant Analysis of Principal Components (DAPC) results based on linguistic groups (a) and geographic groups (b).

**Supplementary Figure S3** MDS plots, based on the *Φ_st_* distance matrix for 73 populations, of dimension 1 vs. 2 (a), dimension 1 vs. 3 (b) and dimension 2 vs. 3 (c). Red, black and dark blue symbols indicate AA, TK and ST populations, respectively. The stress value is 0.0711. Population abbreviations are shown in Supplementary Table S1.

**Supplementary Figure S4** Pairwise *Φ_st_* values with *P*-values obtained by 1000 permutations. Population abbreviations are in Supplementary Table S1.

**Supplementary Figure S5** MDS plot based on the *Φ_st_* distance matrix for 134 populations. The stress value is 0.0961. Symbols represent linguistic families: ▲ = Sino-Tibetan, ◼ = Tai-Kadai, ⚫ = Austroasiatic, ◆ = Austronesian, 🗷 = Dravidian and Indo-European. Black and red indicate Thai/Lao and ISEA populations, respectively while pink and green represent Indian and Chinese populations. Yellow indicates populations from Myanmar, Cambodia and Vietnam. Population abbreviations are shown in Supplementary Table S1.

**Supplementary Figure S6** Networks of major haplogroups.

**Supplementary Figure S7** Bayesian skyline plots (BSP) for each individual population. The 95% highest posterior density limits are indicated by the blue shading. Population abbreviations are in Supplementary Table S1.

**Supplementary Figure S8** Evolution of the ABC-RF prior error rate with respect to the number of trees in the forest for the ABC analysis of the origins of Central Thai groups (A) and of the relationships between populations from different MSEA language families (B).

**Supplementary Figure S9** Linear Discriminate Analysis (LDA) plot for the fit between the observed data and the simulated data generated by each model for the origin of Central Thai groups (a) and the relationships of populations from different language families (b).

**Supplementary Figure S10** Posterior distributions of the parameters estimated for the demic diffusion (A) and continuous migration (B) models. The X axis covers the range of the (uniform) prior distributions. Abbreviations are in Supplementary Table S5.

**Supplementary Table S1** Details for the populations used in the comparative analyses.

**Supplementary Table S2** Haplogroup information in all studied samples.

**Supplementary Table S3** Haplogroup frequencies (in percentage) observed in 22 populations. Bold letter indicates new haplogroups which were not found in our previous study of Thai/Lao populations.

**Supplementary Table S4** Random forests confusion matrix and classification error for the ABC analysis of Central Thai origins.

**Supplementary Table S5** Votes assigned to each model by the Random Forest procedure and posterior probability of the selected model in the ABC analysis of Central Thai origins.

**Supplementary Table S6** Parameters estimation for the demic diffusion and continuous migration model in the ABC analysis of Central Thai origins. NcAA is the current effective population size of Austroasiatic populations, NcCT is the current central Thai effective population size, NcDAI is the current effective population size for Southern Chinese populations, M1 is the migration rate to Austroasiatic groups from Central Thai populations and M2 is the migration rate to Central Thai populations from Austroasiatic groups.

**Supplementary Table S7** Random forests confusion matrix and classification error for the ABC analysis of the relationships between populations from different MSEA language families.

**Supplementary Table S8** Votes assigned to each model by the Random Forest procedure and posterior probability of the selected model in the ABC analysis of the relationships between populations from different MSEA language families.

**Supplementary Text**

**Description of Approximate Bayesian Computation (ABC) analyses**

An ABC approach was utilized to test different demographic scenarios concerning the relationships of SEA language families and the origin of central Thai (CT) populations. Employing an ABC methodology allowed us to simulate the evolution of complete mitochondrial sequences, by means of coalescent theory, under different competing models and to select the model that was best able to recreate the variation observed in our populations. The simulations were generated considering prior distributions associated with different model parameters. For the maternal origin of CT populations, we considered the same three demographic scenarios tested in our previous study^1^ for the origins of North/Northeastern Thai and Laos populations: demic diffusion; an endogenous origin (with cultural diffusion of the TK language); and continuous migration (Figure 2). The demic diffusion model postulates a first split of AA-speaking Mon (MO) and Khmer (KH) from the TK-speaking populations (Xishuangbanna Dai and CT) ~3 kya^2^ followed by a later split of CT from Xishuangbanna Dai ~1.2 kya (Figure 2a).^1,3^ The endogenous scenario involves instead an early split of the Xishuangbanna Dai from CT and AA groups, with a later division of CT and AA ~0.8 kya (Figure 2b).^4^ The continuous migration model incorporates the same demographic history as the demic diffusion model, but includes additional gene-flow between AA and CT after first contact (0.8 kya) (Figure 2c).

For all of the models in the CT origin test, we assumed constant population sizes that were allowed to vary among groups, a fixed mutation rate from Fu *et al*.^5^ (4.80 x 10^-7^), and fixed separation times based on historical records. We assigned a uniform prior on the effective population size of the three groups over the interval 1,000-100,000 and on the migration rate for the admixture model between 0.01-0.20. The mtDNA genomes from CT groups (*n* = 210) were generated in the present study, while Mon (MO) sequences consisted of 49 new sequences generated in the present study plus an additional 153 MO and KH sequences reported previously.^1^ The Xishuangbanna Dai sequences were obtained from a previous study.^6^

For testing the genetic relationships of populations from the different SEA language families by analyzing five tree-like demographic histories (Figure 3), we assume expanding population sizes, a fixed mutation rate^5^ (4.80 x 10^-7^), and fixed separation times based on historical records. We assigned a uniform prior distribution on both the current and ancestral effective population sizes over the range 1,000-100,000 and 1,000-50,000, respectively. We combined our Thai/Lao data with selected published mtDNA genomic data as follows: 1,219 TK sequences (present study and previous studies^1,6^), 876 AN sequences^7-11^, 627 AA sequences (present study and previous studies^1,12^) and 440 ST sequences (present study and previous studies^13-16^) (Supplementary Table S1). Due to the uneven sample sizes of these four groups, we simulated 440 sequences for each of the model populations as 440 sequences represents the smallest sample size; thus, the final dataset consists of 1,760 sequences.

Because of the computational cost of simulating a large number of complete mitochondrial sequences, we utilized a novel approach based on a machine learning tool called “Random Forests”.^17-18^ This new method can greatly reduce the number of simulations required to select the corrected model from a set of competing ones. ABC- Random Forests uses a machine-learning algorithm (based on a reference table of simulations) to predict the most suitable model at each possible value of a set of covariates (i.e. all summary statistics used to summarize the data), and a classification algorithm which allows one to overcome the difficulties in the choice of the summary statistics, while also gaining a larger discriminative power among the competing models (see details in Pudlo *et al*.^17^).

To generate the simulated datasets, we used the software package ABCtoolbox^19^ running 10,000 simulations for each model. We computed a set of summary statistics using arlsumstat^20^ describing both within-population (number of haplotypes, haplotype diversity, total and private number of segregating sites, average number of pairwise differences for each population and Tajima's D)^21-23^, and between-population diversity (*Φ_st_* and mean number of pairwise differences between populations)^24-25^. We randomly resampled 440 sequences from AA, AN and TK groups before computing the summary statistics for the observed data, so as to make them comparable with the simulated data.

The ABC model choice was conducted with the R package *abcrf* ^17^. We built the reference table using the function *abcrf* and employing a forest of 500 trees, as this number was suggested to provide the best trade-off between computational efficiency and statistical precision^17^. However, we checked that this number was sufficient by plotting the estimated values of the prior error rate as a function of the number of trees in the forest using the function *err.abcrf* (Supplementary Figure S8)*.* We also made sure that our set of models were able to reconstruct the variability found in the observed mitochondrial sequences and that, inside each set, we were able to discriminate between different scenarios. In order to achieve these goals we plotted the reference table on the LDA axes using the function *plot.abcrf* and calculated the classification error using as PODs each dataset of our reference table. We carried out the actual model comparisons and obtained the posterior probabilities of the winning models using the function *predict.* As both the demic diffusion and continuous migration models resulted difficult to distinguish (Supplementary Table S3) and received a similar amount of votes in our model selection procedure (Supplementary Table S4), we estimated the parameters involved in these scenarios. We ran additional simulations (200,000) for both models and applied the script available at:

<http://code.google.com/p/popabc/source/browse/#svn%2Ftrunk%2Fscripts>.

When estimating model parameters we reduced the dimensionality of the space of statistics via Partial Least Square (PLS) transformation.^19^ Under this approach, we defined a set of orthogonal linear- combinations of summary statistics best explaining the variance in the model parameter space. After the inspection of the Root Mean Square Error Plots, we selected 10 PLS to calculate the posterior probabilities of model parameters.

**References**

1. Kutanan W, Kampuansai J, Srikummool M, *et al*. Complete mitochondrial genomes of Thai and Lao populations indicate an ancient origin of Austroasiatic groups and demic diffusion in the spread of Tai–Kadai languages. *Hum Genet* 2017; **136**: 85–98.

2. Sun H, Zhou C, Huang X, *et al*. Autosomal STRs provide genetic evidence for the hypothesis that Tai people originate from Southern China. *PLoS ONE* 2013; **8**: e60822.

3. Pittayaporn P. Layers of Chinese loanwords in proto-southwestern Tai as evidence for the dating of the spread of southwestern Tai. *Manusya J Humanit* 2014; **20**: 47–68.

4. Baker C, Phongpaichit P (eds). *A history of Thailand*, 2nd edn. Cambridge University Press: Cambridge, UK, 2009.

5. Fu Q, Mittnik A, Johnson PL, *et al*. A revised timescale for human evolution based on ancient mitochondrial genomes. *Curr Biol* 2013; **23**: 553–559.

6. Diroma MA, Calabrese C, Simone D, *et al*. Extraction and annotation of human mitochondrial genomes from 1000 Genomes Whole Exome Sequencing data. *BMC Genom* 2014; **15**: S2.

7. Gunnarsdottir ED, Li M, Bauchet M, Finstermeier K, Stoneking M. High-throughput sequencing of complete human mtDNA genomes from the Philippines. *Genome Res* 2011a; **21**: 1–11.

8. Gunnarsdóttir ED, Nandineni MR, Li M, *et al*. Larger mitochondrial DNA than Y-chromosome differences between matrilocal and patrilocal groups from Sumatra. *Nat Commun* 2011b; **2**: 228.

9. Jinam TA, Hong LC, Phipps ME, *et al*. Evolutionary history of continental southeast Asians: “early train” hypothesis based on genetic analysis of mitochondrial and autosomal DNA data. *Mol Biol Evol* 2012; **29**: 3513–3527.

10. Ko AMS, Chen CY, Fu Q, *et al*. Early Austronesians: into and out of Taiwan. *Am J Hum Genet* 2014; **94**: 426–436.

11. Delfin FS, Ko AMS, Li M, *et al*. Complete mtDNA genomes of Filipino ethnolinguistic groups: A melting pot of recent and ancient lineages in the Asia-Pacific region. *Eur J Hum Genet* 2014; **22**: 228–237.

12. Zhang X, Qi X, Yang Z, *et al*. Analysis of mitochondrial genome diversity identifies new and ancient maternal lineages in Cambodian aborigines. *Nat Commun* 2013; **4**: 2599.

13. Zhao M, Kong QP, Wang HW, *et al*. Mitochondrial genome evidence reveals successful Late Paleolithic settlement on the Tibetan Plateau. *Proc Natl Acad Sci USA* 2009; **106**: 21230–21235.

14. Zheng HX, Yan S, Qin ZD, *et al*. Major population expansion of East Asians began before Neolithic time: evidence of mtDNA genomes. *PLoS ONE* 2011; **6**: e25835.

15. Summerer M, Horst J, Erhart G, *et al*. Large-scale mitochondrial DNA analysis in Southeast Asia reveals evolutionary effects of cultural isolation in the multi-ethnic population of Myanmar. *BMC Evol Biol* 2014; **14**: 17.

16. Li YC, Wang HW, Tian JY, *et al*. Ancient inland human dispersals from Myanmar into interior East Asia since the Late Pleistocene. *Sci Rep* 2015; **5**: 9473.

17. Pudlo P, Marin JM, Estoup A, Cornuet JM, Gautier M, Robert CP. Reliable ABC model choice via random forests. *Bioinformatics* 2016; **32**: 859–866.

18. Breiman L. Random forests. *Machine learning* 2001; **45**: 5–32.

19. Wegmann D, Leuenberger C, Neuenschwander S, Excoffier L. ABCtoolbox: a versatile toolkit for approximate Bayesian computations. *BMC Bioinformatics* 2010; **11**: 116.

20. Excoffier L, Lischer HEL. Arlequin suite ver 3.5: a new series of programs to perform population genetics analyses under Linux and Windows. *Mol Ecol Resour* 2010; **10**: 564–567.

21. Tajima F. Evolutionary relationship of DNA sequences in finite populations. *Genetics* 1983; **105**: 437–460.

22. Tajima F. Measurement of DNA polymorphism. In: Takahata N, Clark AG (eds). *Mechanisms of Molecular Evolution. Introduction to Molecular Paleopopulation Biology*. Sinauer Associates Inc: Maryland, USA, 1993, pp 37–59.

23. Tajima F. Statistical method for testing the neutral mutation hypothesis by DNA polymorphism. *Genetics* 1989; **123**: 585–595.

24. Weir BS, Cockerham CC. Estimating F‐statistics for the analysis of population structure. *Evolution* 1984; **38**: 1358–1370.

25. Michalakis Y, Excoffier L. A generic estimation of population subdivision using distances between alleles with special reference for microsatellite loci. *Genetics* 1996; **142**: 1061–1064.

**Additional details on mtDNA haplogroups**

There are many lineages with ages older than 30 kya found in our Thai/Lao samples, e.g. B4, B5, D, F1, F3, M7, M*, M12, M13, M17, M21, M71, M73, M74, M91, R9, R22, N10 and U. Many of them are major lineages and distributed in our Thai/Lao samples as well as in other SEA populations, and have been previously discussed.^1^ Here, we focus on some uncommon ancient lineages, i.e. M*, M17, M21, M71, M73, M91 and U. Nineteen sequences were classified as superhaplogroup M* (i.e., they could not be classified into other M sublineages) and date to ~54.27 kya; most of them occur in the Mon (52.63%) and Karen (KPA) (15.79%). M17 bifurcated to M17a and M17c ~40.90 kya, and 61.11% of the M17 lineages are found in Central Thai (CT) groups. M17a is proposed to be an early mtDNA lineage that originated in MSEA and migrated to ISEA,^2-3^ while M17c was previously found in the Philippine populations.^4^ We here date the variation within these lineages to ~29.02 kya (M17a) and ~32.18 kya (M17c) (Table 3). M21 bifurcates ~42.73 kya to (M21b and M21a, with ages for the variation within each clade of 34.54 kya and 3.93 kya, respectively. M21b was found in AA-speaking and CT groups whereas M21a is new lineage in Thai/Lao populations, found in the Karen and MO7. M21a is most common among the Semang and M21b is found in both the Semang and Senoi from Malaysia.^5^ Two major sublineages of M71 are M71(151T) and M71a. Although M71 is rare (~0.02%) in our study, its frequency is higher than reported previously in MSEA^6,7-8^ and ISEA.^4^ The estimated divergence time of M71 is ~31.22 kya, slightly lower than a previous estimate of ~39.40 kya.^7^ The variation within M71(151T) and M71a are ~23.56 kya and ~24.00 kya, respectively. About 50% of M71a is from CT individuals, with the remainder found in other TK groups and in the Blang, an AA group. M73 was mostly contributed by the MO (44.44%) and CT (44.44%). It was also reported previously at low frequency in MSEA^6, 7-8^ and ISEA.^4^ We dated this lineage to ~36.21 kya, consistent with a previous estimate of ~37.80 kya.^7^ Notably, M17, M21, M71 and M73 are ancient maternal lineages of SEA found in both MSEA and ISEA, reflecting linkages between the early lineages in SEA.^9^

M91, dated to ~35.98 kya, is another proposed indigenous SEA haplogroup. The age estimated here is slightly lower than in a previous study of sequences from Myanmar (~39.55 kya).^10^ A sublineage, M91a, dates to ~15.87 kya and is found in MO, Karen (KPA) and CT (Supplementary Table S2). Interestingly, haplogroup U is the second oldest lineage in this study with an age of ~52.60 kya, which is slightly higher than a recent estimate of 49.60 kya.^11^ Subhaplogroups U1 and U2, which are restricted to CT groups, are autochthonous to the Near East^12^ and South Asia^13^, respectively. In summary, the CT groups contrast with other Thai/Lao groups in exhibiting several ancient haplogroups (especially basal M lineages) at low frequency.

**References**

1. Kutanan W, Kampuansai J, Srikummool M, *et al*. Complete mitochondrial genomes of Thai and Lao populations indicate an ancient origin of Austroasiatic groups and demic diffusion in the spread of Tai–Kadai languages. *Hum Genet* 2017; **136**: 85–98.

2. Bellwood P (eds). First Islanders: *Prehistory and Human Migration in* Island *Southeast Asia*. 1st edn. John Wiley & Sons: NJ, USA, 2017.

3. Tumonggor MK, Karafet TM, Hallmark B, *et al*. The Indonesian archipelago: An ancient genetic highway linking Asia and the Pacific. *J Hum Genet* 2013; **58**: 165–173.

4. Tabbada KA. Trejaut J, Loo JH, *et al*. Philippine Mitochondrial DNA Diversity: A Populated Viaduct between Taiwan and Indonesia? *Mol Biol Evol* 2010; **27**: 21-31.

5. Hill C, Soares P, Mormina M, *et al*. Phylogeography and ethnogenesis of aboriginal Southeast Asians. *Mol Biol Evol* 2006; **23**: 2480–2491.

6. Zhang X, Qi X, Yang Z, *et al*. Analysis of mitochondrial genome diversity identifies new and ancient maternal lineages in Cambodian aborigines. *Nat Commun* 2013; **4**: 2599.

7. Peng MS, Quang HH, Dang KP, *et al*. Tracing the Austronesian footprint in mainland Southeast Asia: a perspective from mitochondrial DNA. *Mol Biol Evol* 2010; **27**: 2417–2430.

8. Bodner M, Zimmermann B, Röck A, *et al*. Southeast Asian diversity: first insights into the complex mtDNA structure of Laos. *BMC Evol Biol* 2011; **11**:49.

9. Jinam TA, Hong LC, Phipps ME, *et al*. Evolutionary history of continental southeast Asians: “early train” hypothesis based on genetic analysis of mitochondrial and autosomal DNA data. *Mol Biol Evol* 2012; **29**: 3513-3527.

10. Li YC, Wang HW, Tian JY, *et al*. Ancient inland human dispersals from Myanmar into interior East Asia since the Late Pleistocene. *Sci Rep* 2015; **5**: 9473.

11. Larruga JM, Marrero P, Abu-Amero KK, Golubenko MV, Cabrera VM. Carriers of mitochondrial DNA macrohaplogroup R colonized Eurasia and Australasia from a southeast Asia core area. *BMC Evol Biol* 2017; **17**: 115.

12. Derenko M, Malyarchuk B, Bahmanimehr A, *et al*. Complete Mitochondrial DNA Diversity in Iranians. *PLoS ONE* 2013; **8**: e80673.

13. Palanichamy MG, Sun C, Agrawal S, *et al*. Phylogeny of Mitochondrial DNA Macrohaplogroup N in India, Based on Complete Sequencing: Implications for the Peopling of South Asia. *Am J Hum Genet* 2004; **75**: 966-978.

**
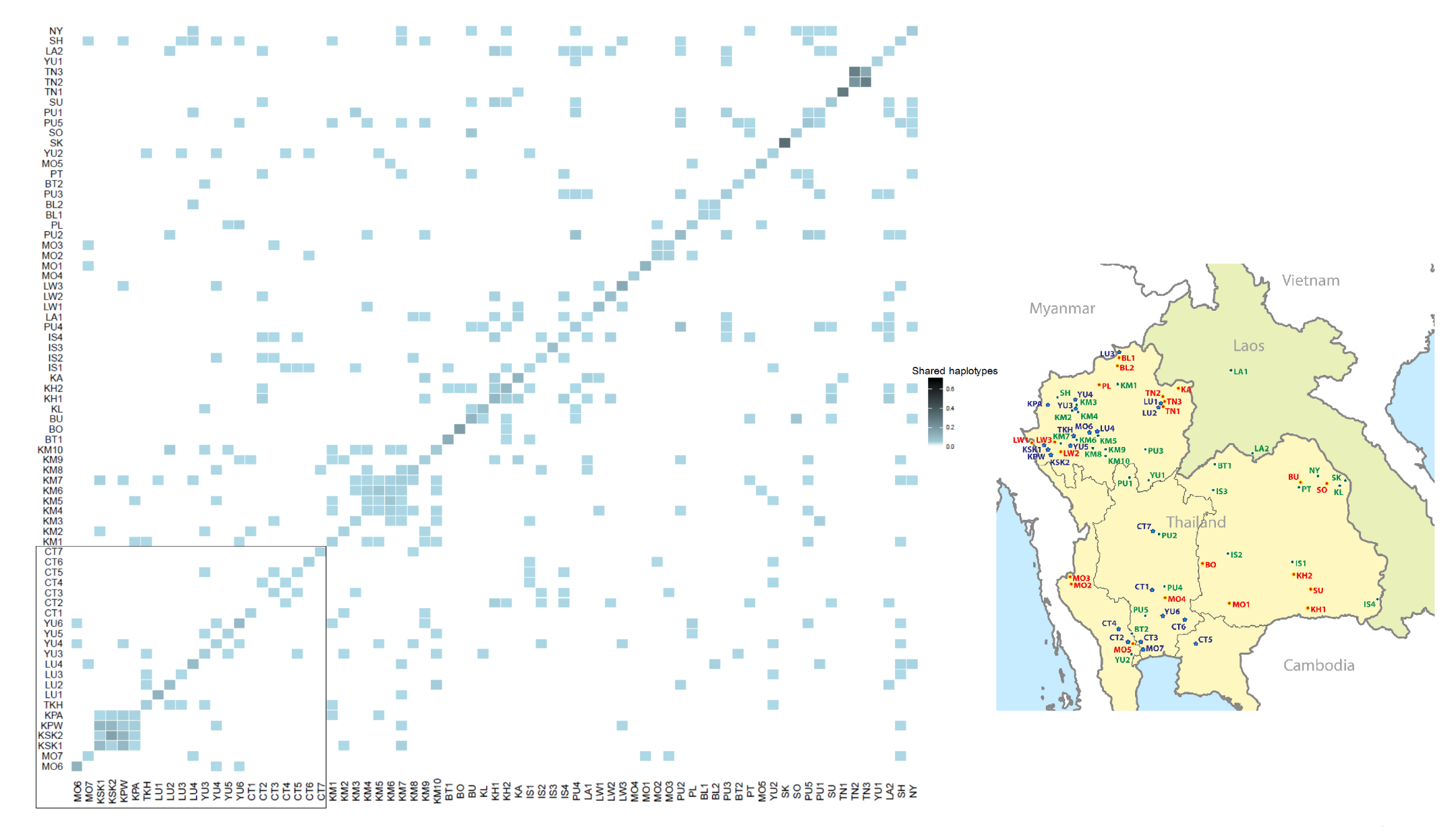
**

**Supplementary Figure S1** Relative frequency of shared haplotypes within and among 73 Thai/Lao populations. Population abbreviations are in Supplementary Table S1.

(a)

(b)

**Supplementary Figure S2** Discriminant Analysis of Principal Components (DAPC) results based on linguistic groups (a) and geographic groups (b). Colors in (a) represent Tai-Kadai (green), Sino-Tibetan (orange) and Austroasiatic (light purple) populations. Colors in (b) represent Northern (green), Northeastern (orange), Central (light purple) and Western (pink) Thai populations.


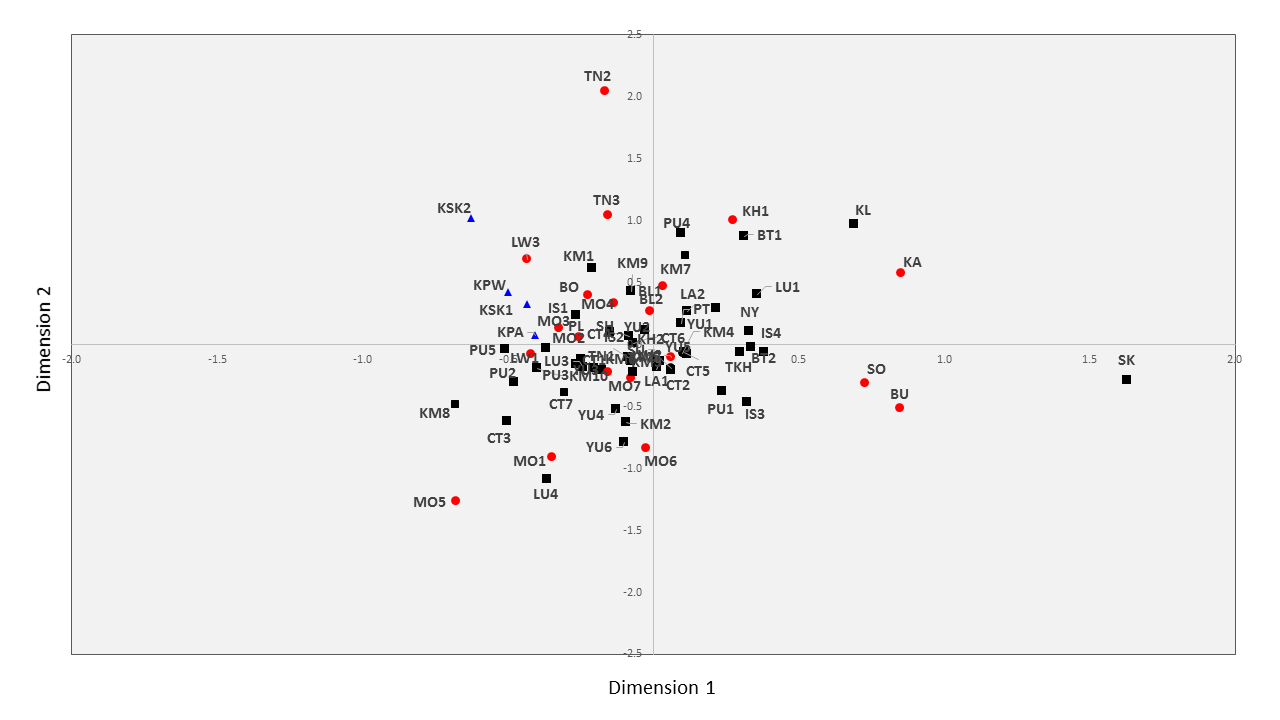


(a)


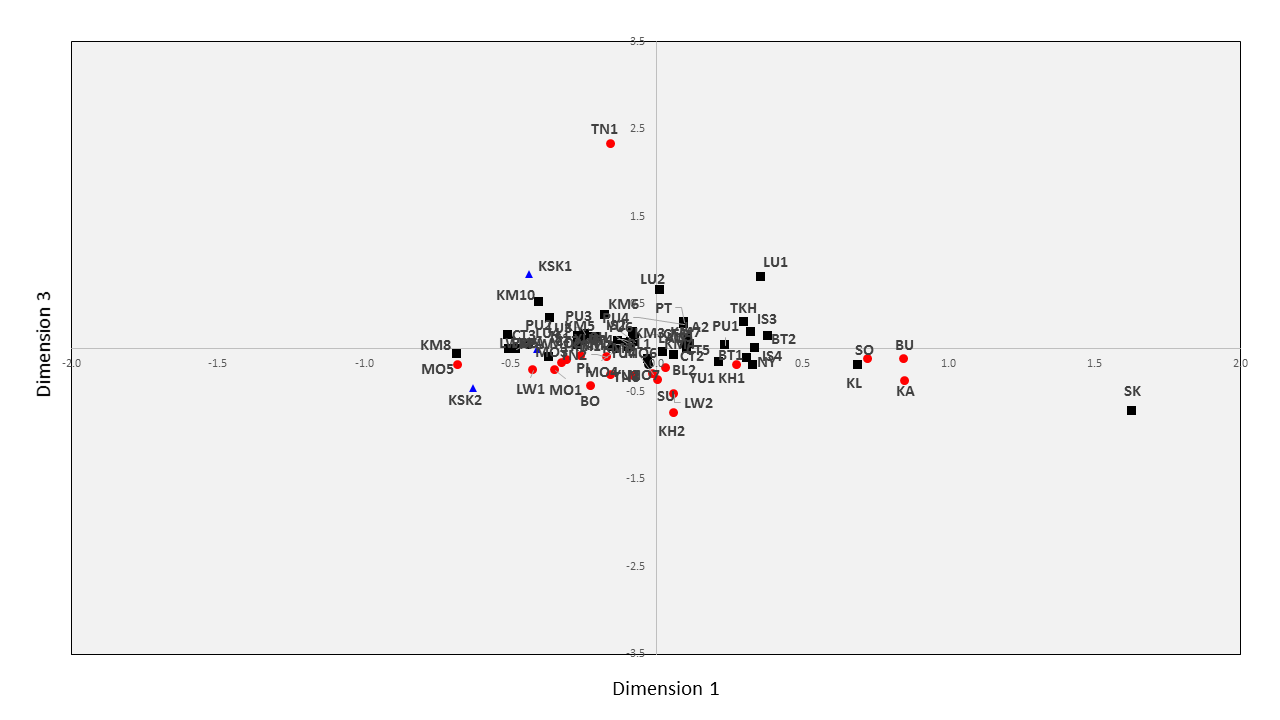
(b)


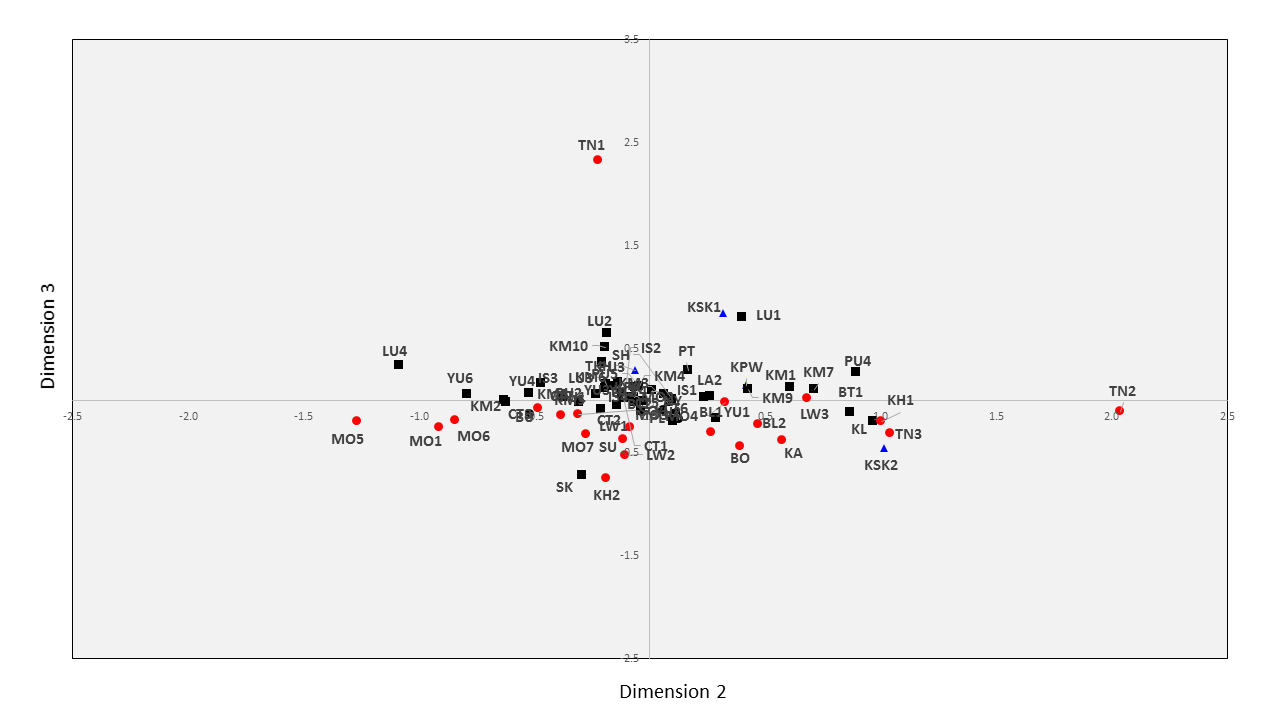
(c)

**Supplementary Figure S3** The MDS plot based on the *Φ_st_* distance matrix for 73 populations: dimension 1 vs. 2 (a), dimension 1 vs. 3 (b) and dimension 2 vs. 3 (c). Red, black and dark blue symbols indicate AA, TK and ST populations, respectively. The stress value is 0.0711. Population abbreviations are shown in Supplementary Table S1.

**
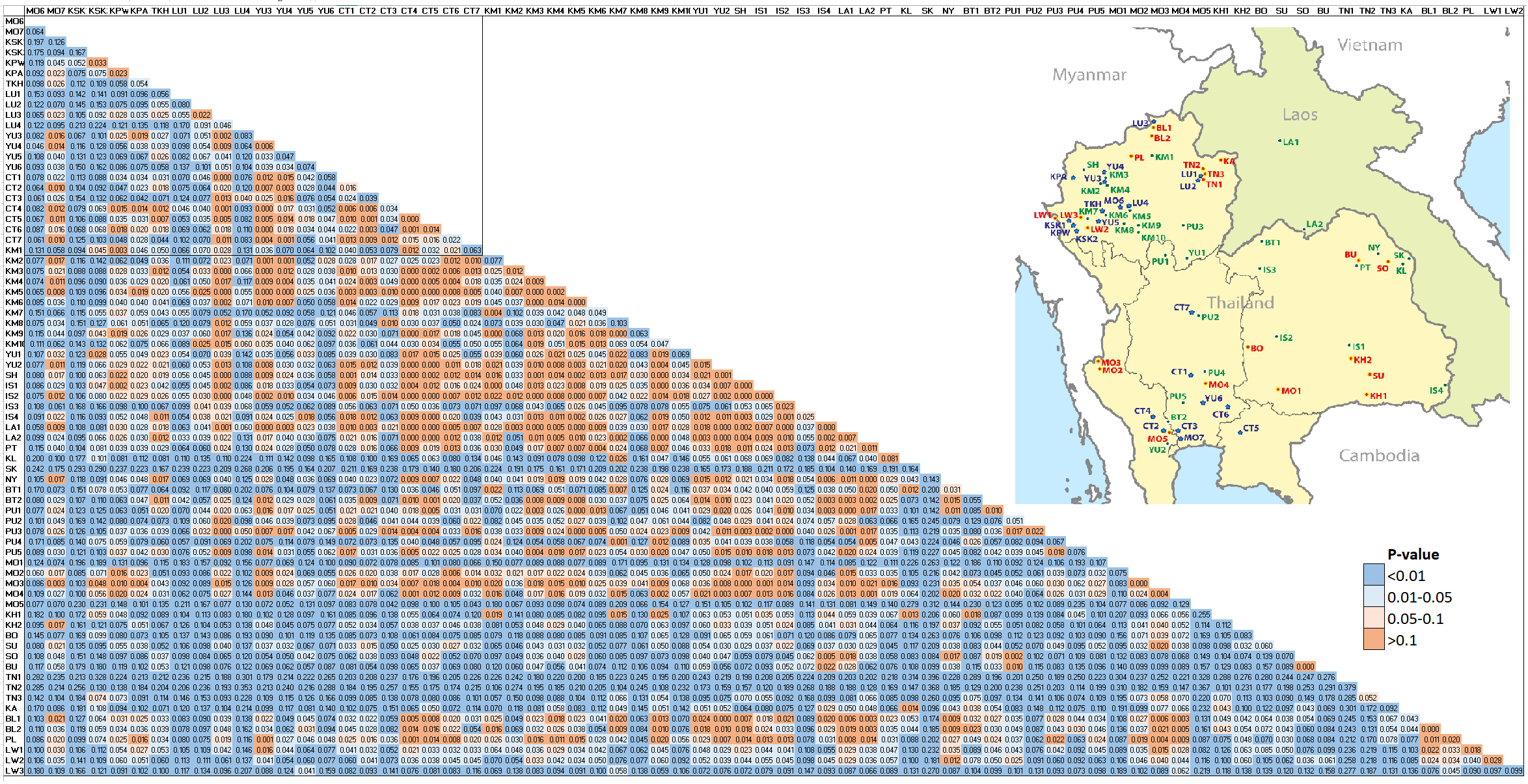
**

**Supplementary Figure S4** Pairwise *Φ_st_* with *P*-values executed by 1,000 permutations. Population abbreviations are in Supplementary Table S1.


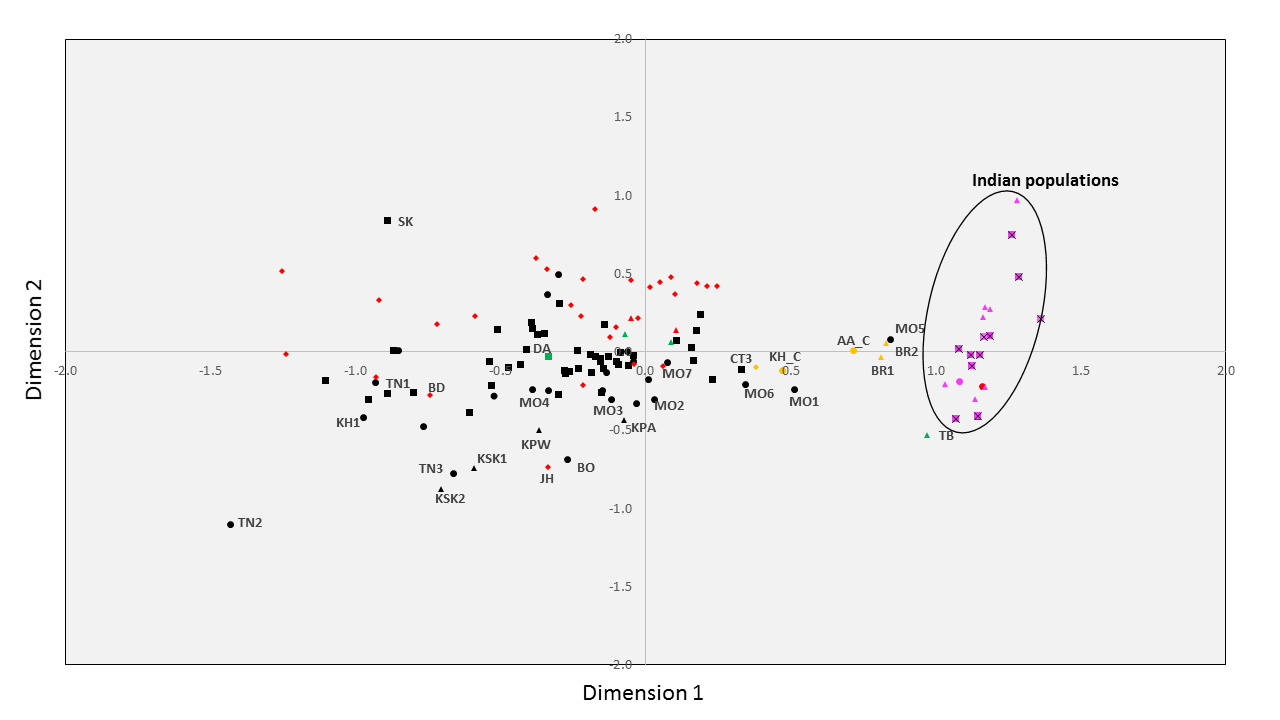


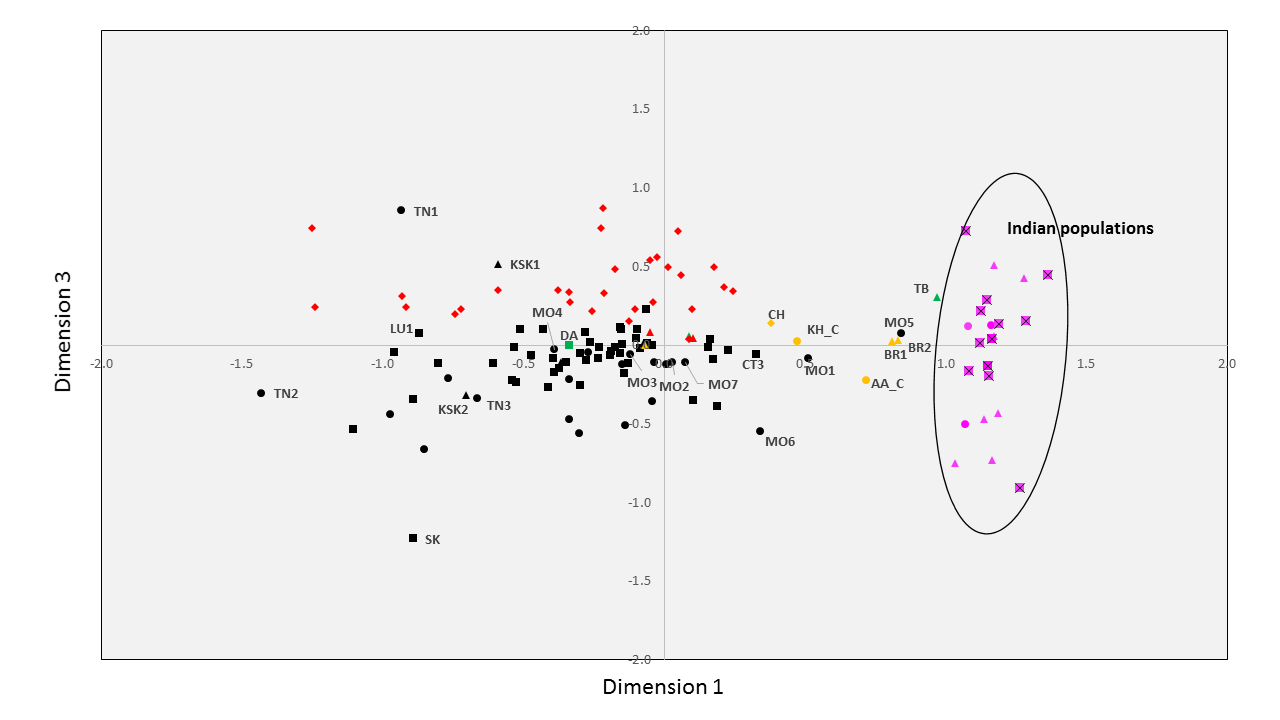


**Supplementary Figure S5** The MDS plot based on the *Φ_st_* distance matrix for 134 populations. The stress value is 0.0961. Symbols indicate linguistic family: ▲ = Sino-Tibetan, ◼ = Tai-Kadai, ⚫ = Austroasiatic, ◆ = Austronesian, 🗷 = Dravidian and Indo-European. Black and red indicate Thai/Lao and ISEA populations, respectively while pink and green represent Indian and Chinese populations. Yellow indicates populations from Myanmar, Cambodia and Vietnam. Population abbreviations are shown in Supplementary Table S1.

**
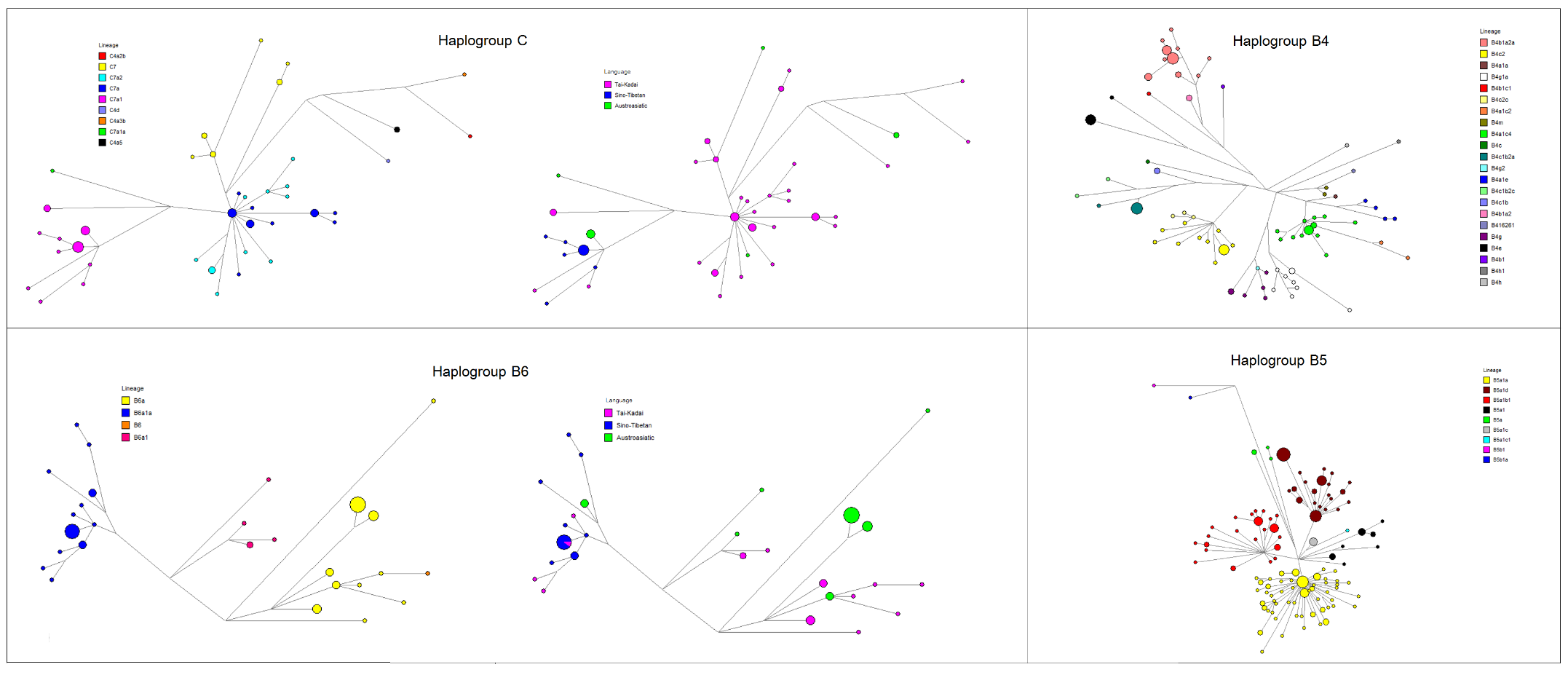
**

**
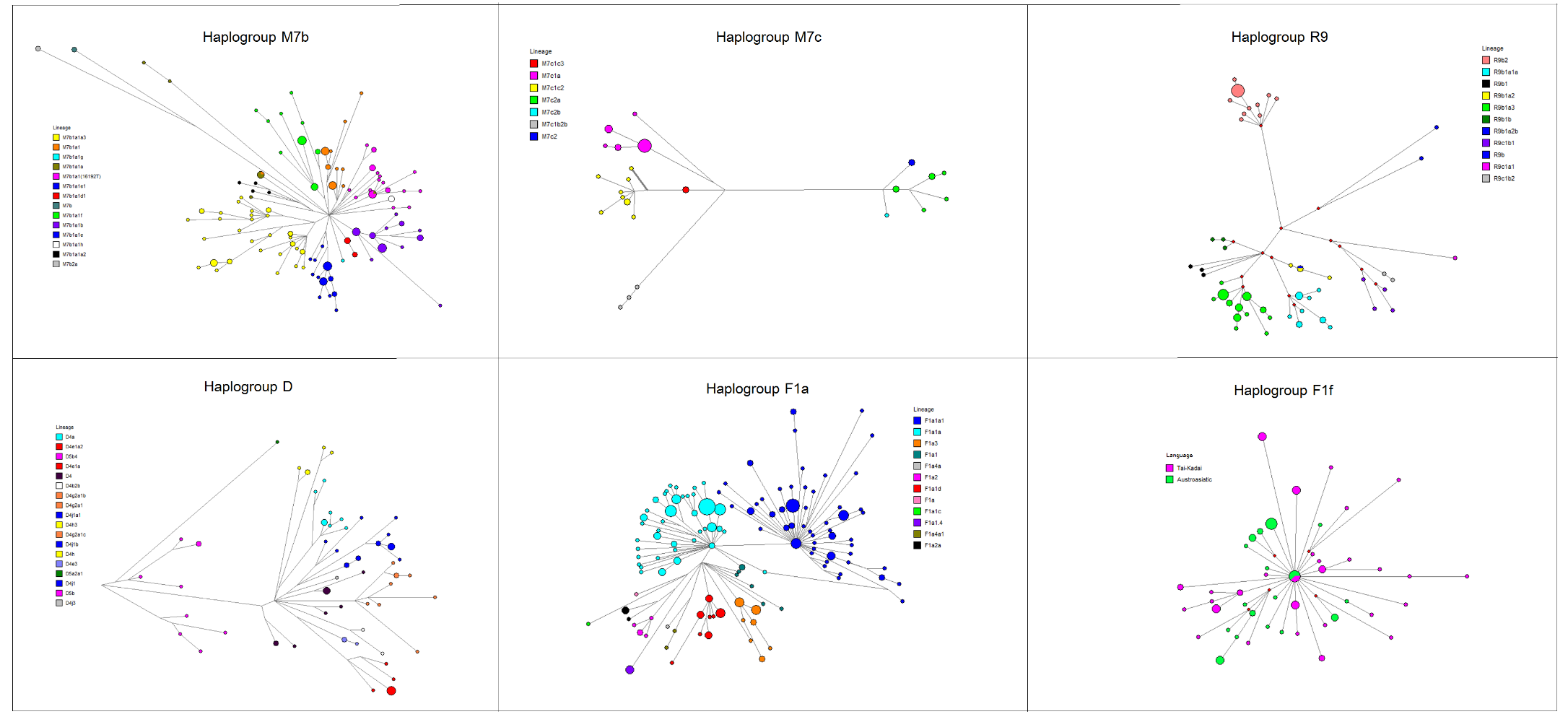
**

**Supplementary Figure S6** Networks of major haplogroups.

Mon (MO6) Mon (MO7)

Karen (KSK1) Karen (KSK2)

Karen (KPW) Karen (KPA)

Khuen (TKH) Lue (LU1)

Lue (LU2) Lue (LU3)

Lue (LU4) Yuan (YU3)

Yuan (YU4) Yuan (YU5)

Yuan (YU6) Central Thai (CT1)

Central Thai (CT2) Central Thai (CT3)

Central Thai (CT4) Central Thai (CT5)


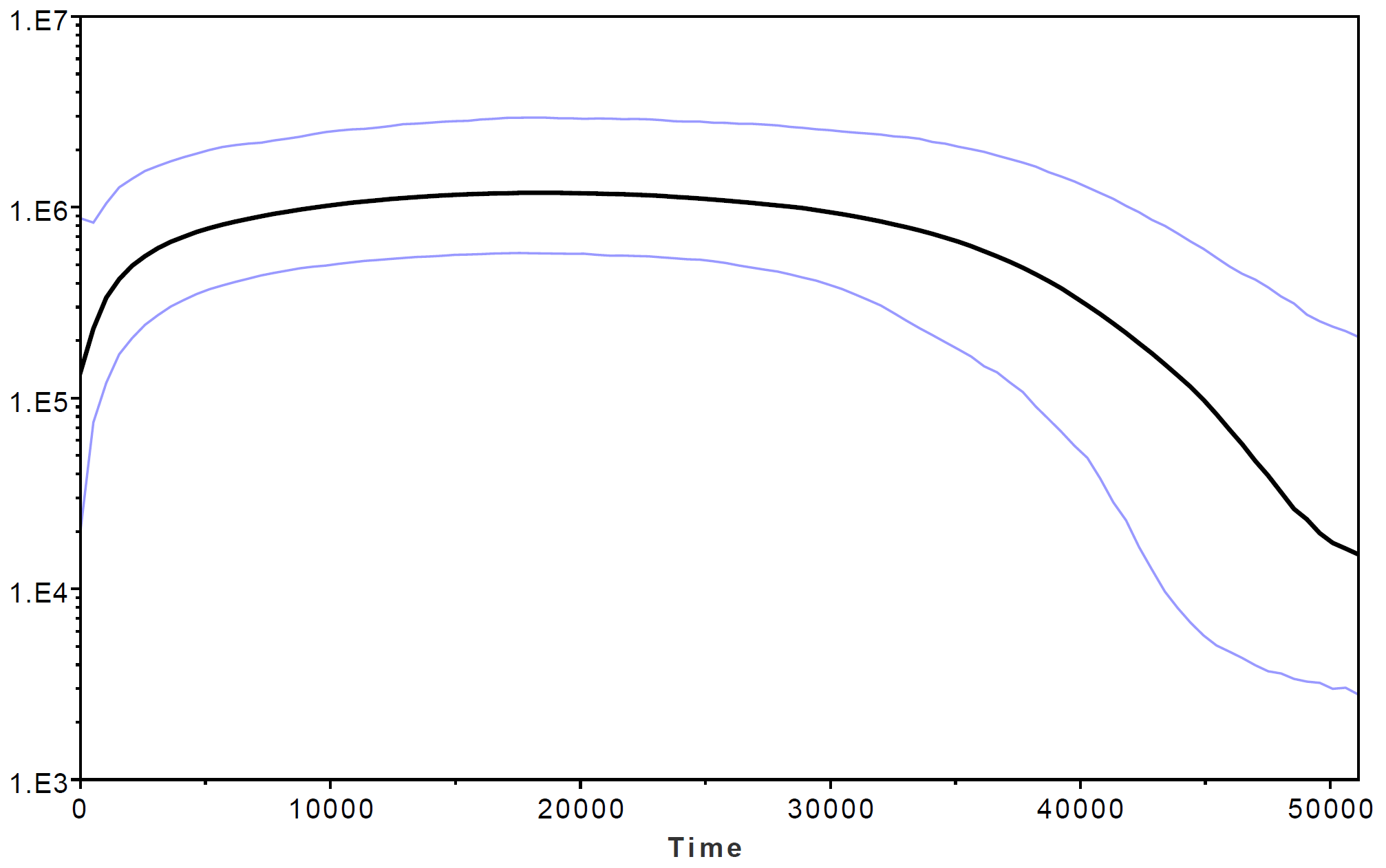


Central Thai (CT6) Central Thai (CT7)

**Supplementary Figure S7** Bayesian skyline plots (BSP) for each individual population. The 95% highest posterior density limits are indicated by the blue lines.


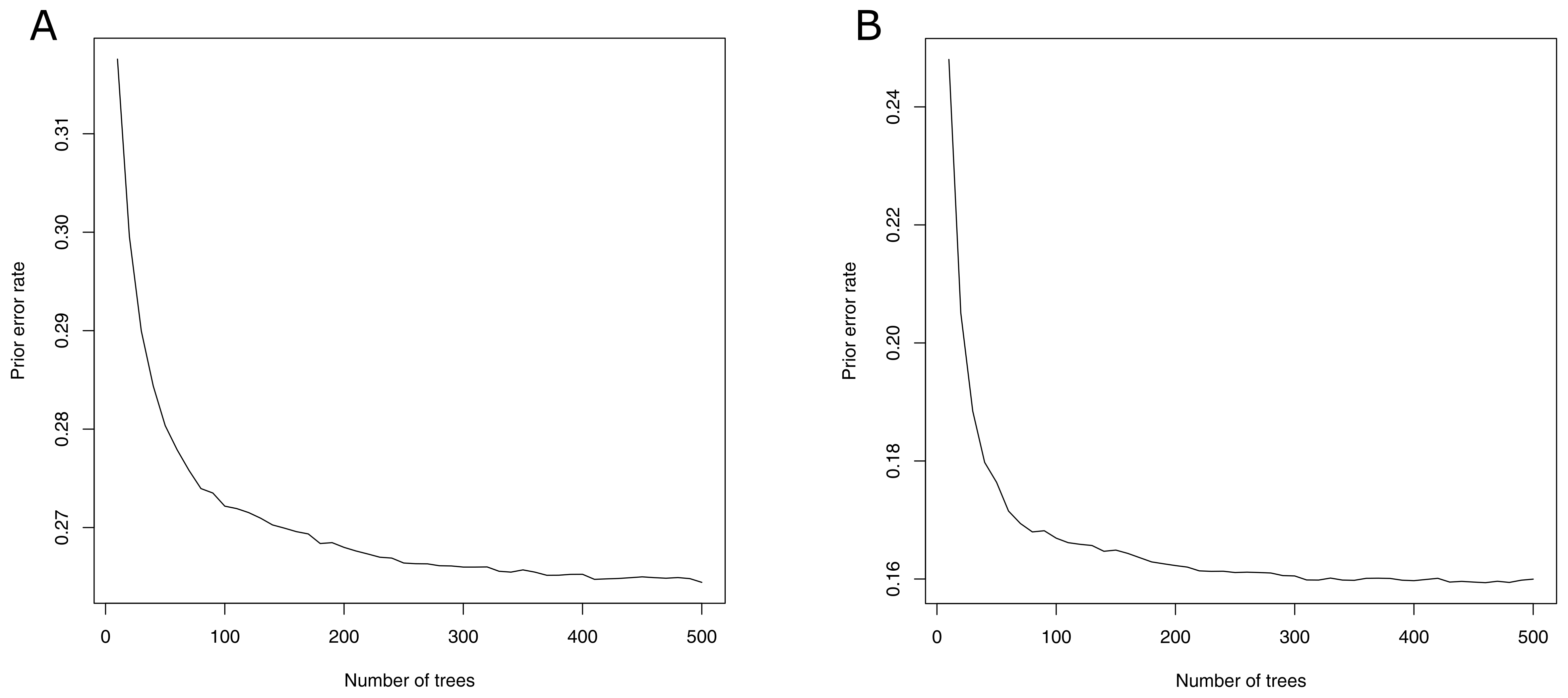


**Supplementary Figure S8** Evolution of the ABC-RF prior error rate with respect to the number of trees in the forest for the ABC analysis on Central Thai origin (A) and on the relationships between populations from different MSEA language families (B)


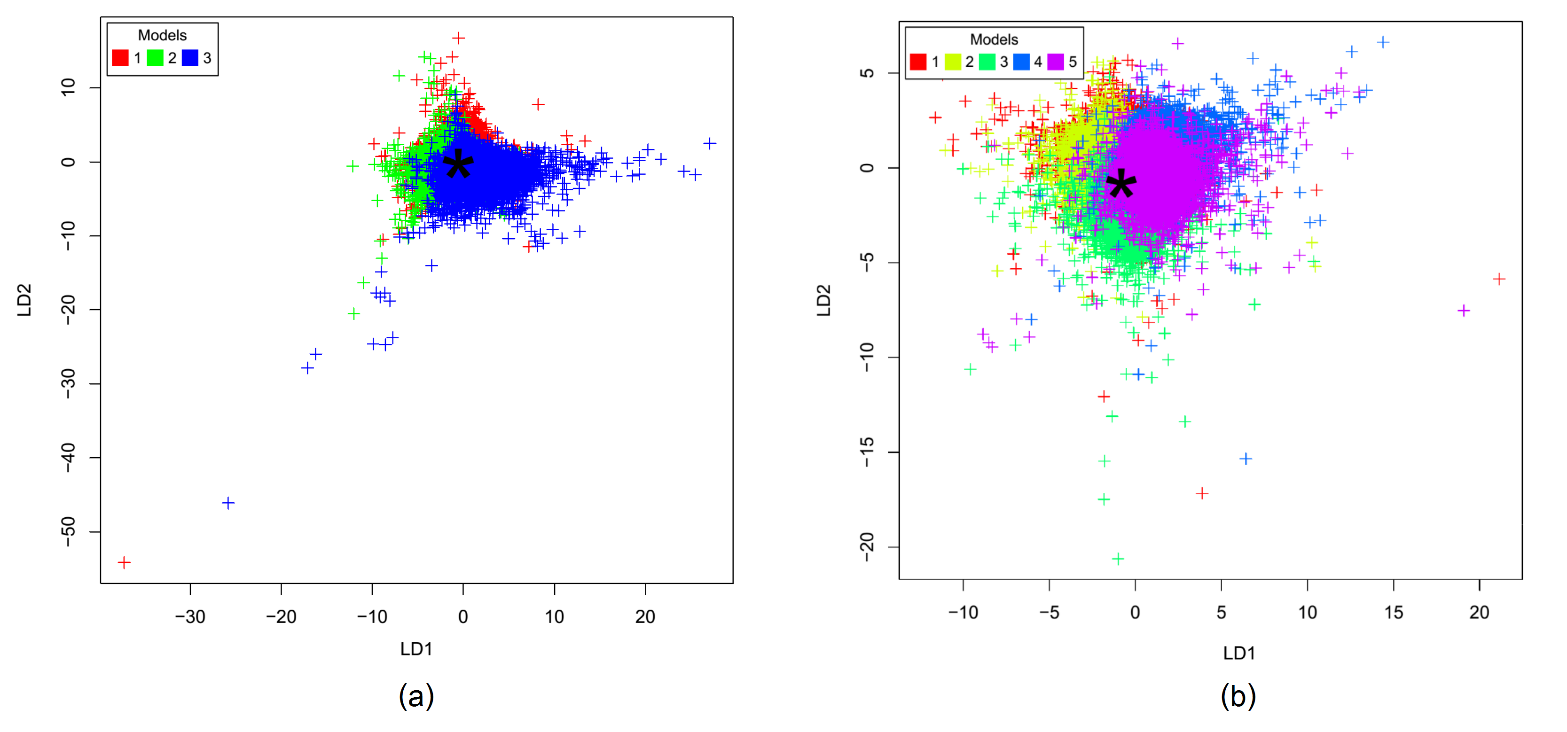


**Supplementary Figure S9** Linear Discriminate Analysis (LDA) plot for the fit between the observed data and the simulated data generated by each model for the origin of Central Thai groups (a) the relationships between populations from different MSEA language families (b).


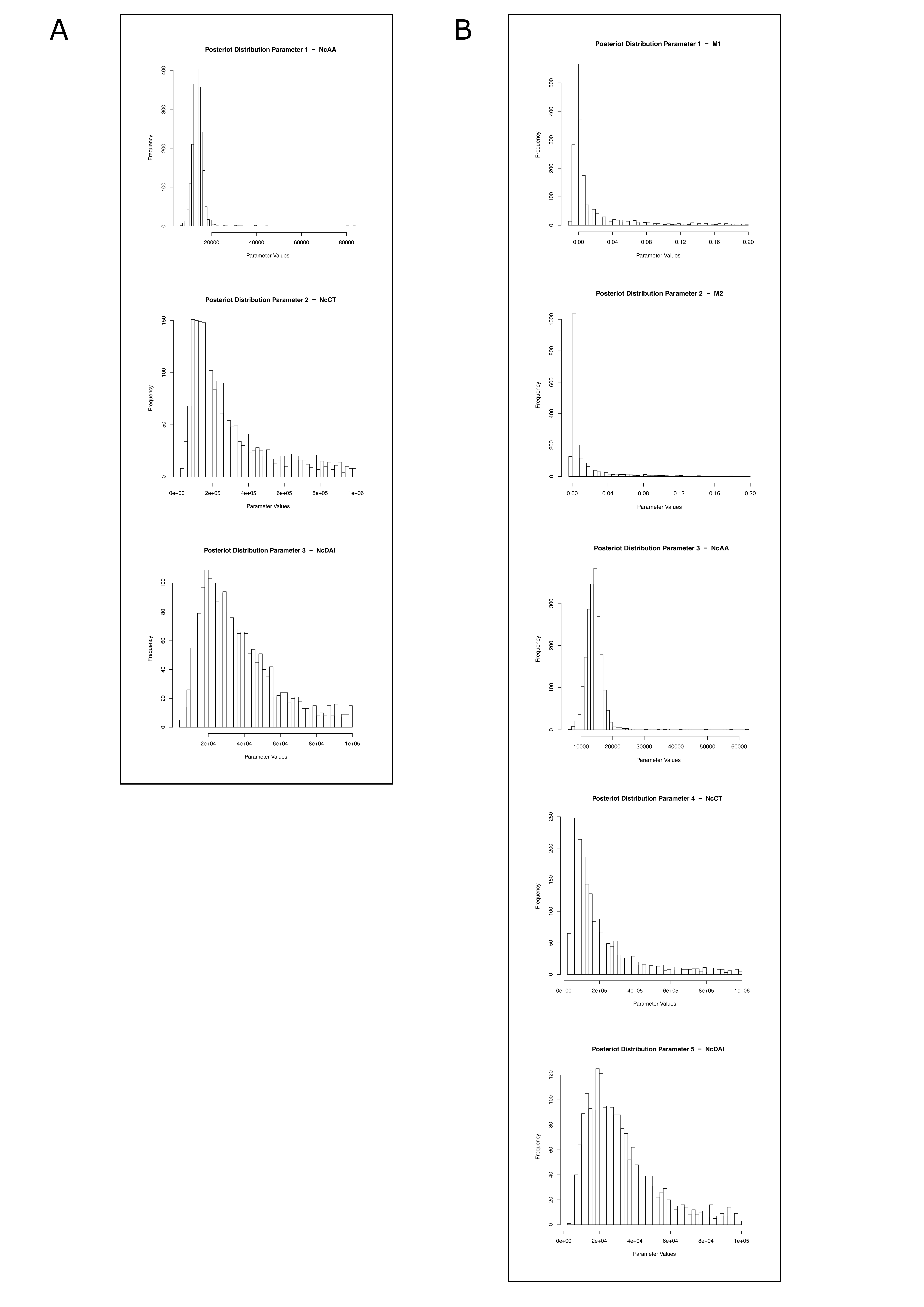


**Supplementary Figure S10** Posterior distributions of the parameters estimated for the demic diffusion (A) and continuous migration (B) models**.** The X axis covers the range of the (uniform) prior distributions. Abbreviations are in Supplementary Table S5.

**Supplementary Table S1** Details for populations in the comparative analyses.

| **Citation** | **Population** | **Language** | **Country** | **Sample size** | **Code** |
| --- | --- | --- | --- | --- | --- |
| Present study | Mon | Austroasiatic | Thailand | 24 | MO6 |
| Present study | Mon | Austroasiatic | Thailand | 25 | MO7 |
| Present study | Karen | Sino-Tibetan | Thailand | 25 | KSK1 |
| Present study | Karen | Sino-Tibetan | Thailand | 13 | KSK2 |
| Present study | Karen | Sino-Tibetan | Thailand | 24 | KPW |
| Present study | Karen | Sino-Tibetan | Thailand | 25 | KPA |
| Present study | Khuen | Tai-Kadai | Thailand | 25 | TKH |
| Present study | Lue | Tai-Kadai | Thailand | 25 | LU1 |
| Present study | Lue | Tai-Kadai | Thailand | 23 | LU2 |
| Present study | Lue | Tai-Kadai | Thailand | 25 | LU3 |
| Present study | Lue | Tai-Kadai | Thailand | 16 | LU4 |
| Present study | Yuan | Tai-Kadai | Thailand | 25 | YU3 |
| Present study | Yuan | Tai-Kadai | Thailand | 25 | YU4 |
| Present study | Yuan | Tai-Kadai | Thailand | 26 | YU5 |
| Present study | Yuan | Tai-Kadai | Thailand | 25 | YU6 |
| Present study | CentralThai | Tai-Kadai | Thailand | 30 | CT1 |
| Present study | CentralThai | Tai-Kadai | Thailand | 30 | CT2 |
| Present study | CentralThai | Tai-Kadai | Thailand | 30 | CT3 |
| Present study | CentralThai | Tai-Kadai | Thailand | 30 | CT4 |
| Present study | CentralThai | Tai-Kadai | Thailand | 30 | CT5 |
| Present study | CentralThai | Tai-Kadai | Thailand | 29 | CT6 |
| Present study | CentralThai | Tai-Kadai | Thailand | 31 | CT7 |
| Kutanan *et al*.^1^ | KhonMueang | Tai-Kadai | Thailand | 25 | KM1 |
| Kutanan *et al*.^1^ | KhonMueang | Tai-Kadai | Thailand | 25 | KM2 |
| Kutanan *et al*.^1^ | KhonMueang | Tai-Kadai | Thailand | 24 | KM3 |
| Kutanan *et al*.^1^ | KhonMueang | Tai-Kadai | Thailand | 25 | KM4 |
| Kutanan *et al*.^1^ | KhonMueang | Tai-Kadai | Thailand | 23 | KM5 |
| Kutanan *et al*.^1^ | KhonMueang | Tai-Kadai | Thailand | 25 | KM6 |
| Kutanan *et al*.^1^ | KhonMueang | Tai-Kadai | Thailand | 25 | KM7 |
| Kutanan *et al*.^1^ | KhonMueang | Tai-Kadai | Thailand | 25 | KM8 |
| Kutanan *et al*.^1^ | KhonMueang | Tai-Kadai | Thailand | 24 | KM9 |
| Kutanan *et al*.^1^ | KhonMueang | Tai-Kadai | Thailand | 25 | KM10 |
| Kutanan *et al*.^1^ | Yuan | Tai-Kadai | Thailand | 17 | YU1 |
| Kutanan *et al*.^1^ | Yuan | Tai-Kadai | Thailand | 25 | YU2 |
| Kutanan *et al*.^1^ | Shan | Tai-Kadai | Thailand | 25 | SH |
| Kutanan *et al*.^1^ | LaoIsan | Tai-Kadai | Thailand | 25 | IS1 |
| Kutanan *et al*.^1^ | LaoIsan | Tai-Kadai | Thailand | 25 | IS2 |
| Kutanan *et al*.^1^ | LaoIsan | Tai-Kadai | Thailand | 25 | IS3 |
| Kutanan *et al*.^1^ | LaoIsan | Tai-Kadai | Thailand | 25 | IS4 |
| Kutanan *et al*.^1^ | Lao | Tai-Kadai | Laos | 25 | LA1 |
| Kutanan *et al*.^1^ | Lao | Tai-Kadai | Laos | 24 | LA2 |
| Kutanan *et al*.^1^ | Phutai | Tai-Kadai | Thailand | 25 | PT |
| Kutanan *et al*.^1^ | Kalueng | Tai-Kadai | Thailand | 25 | KL |
| Kutanan *et al*.^1^ | Seak | Tai-Kadai | Thailand | 26 | SK |
| Kutanan *et al*.^1^ | Nyaw | Tai-Kadai | Thailand | 25 | NY |
| Kutanan *et al*.^1^ | BlackTai | Tai-Kadai | Thailand | 25 | BT1 |
| Kutanan *et al*.^1^ | BlackTai | Tai-Kadai | Thailand | 25 | BT2 |
| Kutanan *et al*.^1^ | Phuan | Tai-Kadai | Thailand | 25 | PU1 |
| Kutanan *et al*.^1^ | Phuan | Tai-Kadai | Thailand | 25 | PU2 |
| Kutanan *et al*.^1^ | Phuan | Tai-Kadai | Thailand | 25 | PU3 |
| Kutanan *et al*.^1^ | Phuan | Tai-Kadai | Thailand | 25 | PU4 |
| Kutanan *et al*.^1^ | Phuan | Tai-Kadai | Thailand | 25 | PU5 |
| Kutanan *et al*.^1^ | Mon | Austroasiatic | Thailand | 25 | MO1 |
| Kutanan *et al*.^1^ | Mon | Austroasiatic | Thailand | 23 | MO2 |
| Kutanan *et al*.^1^ | Mon | Austroasiatic | Thailand | 15 | MO3 |
| Kutanan *et al*.^1^ | Mon | Austroasiatic | Thailand | 25 | MO4 |
| Kutanan *et al*.^1^ | Mon | Austroasiatic | Thailand | 22 | MO5 |
| Kutanan *et al*.^1^ | Khmer | Austroasiatic | Thailand | 19 | KH1 |
| Kutanan *et al*.^1^ | Khmer | Austroasiatic | Thailand | 25 | KH2 |
| Kutanan *et al*.^1^ | Nyahkur | Austroasiatic | Thailand | 23 | BO |
| Kutanan *et al*.^1^ | Suay | Austroasiatic | Thailand | 25 | SU |
| Kutanan *et al*.^1^ | So | Austroasiatic | Thailand | 25 | SO |
| Kutanan *et al*.^1^ | Bru | Austroasiatic | Thailand | 24 | BU |
| Kutanan *et al*.^1^ | Htin | Austroasiatic | Thailand | 25 | TN1 |
| Kutanan *et al*.^1^ | Htin | Austroasiatic | Thailand | 25 | TN2 |
| Kutanan *et al*.^1^ | Htin | Austroasiatic | Thailand | 25 | TN3 |
| Kutanan *et al*.^1^ | Khamu | Austroasiatic | Thailand | 25 | KA |
| Kutanan *et al*.^1^ | Blang | Austroasiatic | Thailand | 25 | BL1 |
| Kutanan *et al*.^1^ | Blang | Austroasiatic | Thailand | 25 | BL2 |
| Kutanan *et al*.^1^ | Paluang | Austroasiatic | Thailand | 25 | PL |
| Kutanan *et al*.^1^ | Lawa | Austroasiatic | Thailand | 22 | LW1 |
| Kutanan *et al*.^1^ | Lawa | Austroasiatic | Thailand | 24 | LW2 |
| Kutanan *et al*.^1^ | Lawa | Austroasiatic | Thailand | 24 | LW3 |
| Zhang *et al*.^2^ | Khmer | Austroasiatic | Cambodia | 18 | KH_C |
| Zhang *et al*.^2^ | Austroasiatic | Austroasiatic | Cambodia | 65 | AA_C |
| Summerer *et al*.^3^ | Barma | Sino-Tibetan | Myanmar | 20 | BR1 |
| Li *et al*. (2015)^4^ | Barma | Sino-Tibetan | Myanmar | 73 | BR2 |
| Peng *et al*. (2010)^5^ | Cham | Austronesian | Vietnam | 16 | CH |
| Diroma *et al*. (2014)^6^ | Dai | Tai-Kadai | China | 56 | DA |
| Zheng *et al*. (2011)^7^ | Han | Sino-Tibetan | China | 55 | HN_S |
| Zheng *et al*. (2011)^7^ | Han | Sino-Tibetan | China | 89 | HN_N |
| Zhao *et al*. (2009)^8^ | Tibetan | Sino-Tibetan | Tibet and southern China | 23 | TB |
| Jinam *et al*. (2012)^9^ | Temuan | Austronesian | West Malysia | 18 | TM |
| Jinam *et al*. (2012) ^9^ | Seletar | Austronesian | West Malysia | 21 | SE |
| Jinam *et al*. (2012) ^9^ | Jehai | Austronesian | West Malysia | 24 | JH |
| Jinam *et al*. (2012) ^9^ | Bidayuh | Austronesian | Indonesia | 23 | BD |
| Gunnarsdóttir *et al*. (2011b) ^10^ | Semende | Austronesian | Indonesia | 35 | SMD |
| Gunnarsdóttir *et al*. (2011b) ^10^ | Besemah | Austronesian | Indonesia | 36 | BS |
| Gunnarsdottir *et al*. (2011a) ^11^ | Mamanwa | Austronesian | The Philippines | 32 | MM |
| Gunnarsdottir *et al*. (2011a) ^11^ | Manobo | Austronesian | The Philippines | 40 | MAN |
| Gunnarsdottir *et al*. (2011a) ^11^ | Surigaonon | Austronesian | The Philippines | 25 | SR |
| Delfin *et al*. (2014) ^12^ | Abaknon | Austronesian | The Philippines | 30 | AB |
| Delfin *et al*. (2014) ^12^ | Aeta_Bataan | Austronesian | The Philippines | 21 | AEB |
| Delfin *et al*. (2014) ^12^ | Bagalot | Austronesian | The Philippines | 30 | BAG |
| Delfin *et al*. (2014) ^12^ | Ibaloi | Austronesian | The Philippines | 26 | IB |
| Delfin *et al*. (2014) ^12^ | Ifugao | Austronesian | The Philippines | 29 | IF |
| Delfin *et al*. (2014) ^12^ | Ivatan | Austronesian | The Philippines | 29 | IV |
| Delfin *et al*. (2014) ^12^ | Kalangoya | Austronesian | The Philippines | 26 | KAG |
| Delfin *et al*. (2014) ^12^ | Kankanaey | Austronesian | The Philippines | 30 | KAN |
| Delfin *et al*. (2014) ^12^ | Maranao | Austronesian | The Philippines | 18 | MR |
| Ko *et al*. (2014) ^13^ | Saisiat | Austronesian | Taiwan | 24 | SAI |
| Ko *et al*. (2014) ^13^ | Atayal | Austronesian | Taiwan | 50 | ATA |
| Ko *et al*. (2014) ^13^ | Tsou | Austronesian | Taiwan | 48 | TSO |
| Ko *et al*. (2014) ^13^ | Bunun | Austronesian | Taiwan | 51 | BUN |
| Ko *et al*. (2014) ^13^ | Puyuma | Austronesian | Taiwan | 39 | PUY |
| Ko *et al*. (2014) ^13^ | Rukai | Austronesian | Taiwan | 25 | RUK |
| Ko *et al*. (2014) ^13^ | Paiwan | Austronesian | Taiwan | 50 | PAI |
| Ko *et al*. (2014) ^13^ | Ami | Austronesian | Taiwan | 19 | AMI |
| Ko *et al*. (2014) ^13^ | Tao | Austronesian | Taiwan | 25 | TAO |
| Ko *et al*. (2014) ^13^ | Hakka | Sino-Tibetan | Taiwan | 23 | HAK |
| Ko *et al*. (2014) ^13^ | Minnan | Sino-Tibetan | Taiwan | 25 | MIN |
| Ko *et al*. (2014) ^13^ | Makatao | Austronesian | Taiwan | 50 | MAK |
| Chandrasekar *et al*. (2009) ^14^ | Malpaharia | Austroasiatic | East India | 15 | ML |
| Chandrasekar *et al*. (2009) ^14^ | Munda | Austroasiatic | East India | 31 | MUN |
| Chandrasekar *et al*. (2009) ^14^ | Andh | Indo-European | Central India | 19 | AD |
| Chandrasekar *et al*. (2009) ^14^ | Dirang Monpa | Sino-Tibetan | Northeast India | 30 | DR |
| Chandrasekar *et al*. (2009) ^14^ | Dongri Bhill | Indo-European | West India | 43 | DB |
| Chandrasekar *et al*. (2009) ^14^ | Gallong | Sino-Tibetan | Northeast India | 39 | GL |
| Chandrasekar *et al*. (2009) ^14^ | Jenu Kuruba | Dravidian | South India | 79 | JK |
| Chandrasekar *et al*. (2009) ^14^ | Kamar | Indo-European | Central India | 53 | KMR |
| Chandrasekar *et al*. (2009) ^14^ | Kathakur | Indo-European | West India | 19 | KU |
| Chandrasekar *et al*. (2009) ^14^ | Kathodi | Indo-European | West India | 15 | KD |
| Chandrasekar *et al*. (2009) ^14^ | Katkari | Indo-European | West India | 21 | KR |
| Chandrasekar *et al*. (2009) ^14^ | Korku | Austroasiatic | Central India | 15 | KK |
| Chandrasekar *et al*. (2009) ^14^ | Lachungpa | Sino-Tibetan | Northeast India | 25 | LAH |
| Chandrasekar *et al*. (2009) ^14^ | Lepcha | Sino-Tibetan | Northeast India | 20 | LP |
| Chandrasekar *et al*. (2009) ^14^ | Mathakur | Indo-European | West India | 11 | MT |
| Chandrasekar *et al*. (2009) ^14^ | Madia | Dravidian | East India | 20 | MAA |
| Chandrasekar *et al*. (2009) ^14^ | Nihal | Indo-European | Central India | 28 | NI |
| Chandrasekar *et al*. (2009) ^14^ | Pauri Bhuiya | Dravidian/Indo-European | Central India | 32 | PB |
| Chandrasekar *et al*. (2009) ^14^ | Shertukpen | Sino-Tibetan | Northeast India | 15 | ST |
| Chandrasekar *et al*. (2009) ^14^ | Sonowal Kachari | Indo-European | Northeast India | 19 | SOK |
| Chandrasekar *et al*. (2009) ^14^ | Toto | Sino-Tibetan | Northeast India | 28 | TO |
| Chandrasekar *et al*. (2009) ^14^ | Wanchoo | Sino-Tibetan | Northeast India | 22 | WA |

Notes: 1) All populations were used in genetic distance calculations while those used for the model selection ABC analyses are highlighted in yellow.

2) The compared mtDNA sequences datasets from most previous studies were retrieved from the NCBI Genbank database with the following accession numbers; Kutanan *et al*.^1^ (KX456435–KX457668), Zhang *et al*.^2^ (KC887456 to KC887497), Summerer *et al*.^3^ (JX289092-JX289135), Li *et al*. ^4^ (KP345975-KP346066), Peng *et al*.^5^ (GQ301863–GQ301886), Zhao *et al*.^8^ (GU014563-GU014569), Jinam *et al*.^9^ (AP012346–AP012431), Gunnarsdóttir *et al*.^10^ (HM596644 -HM596715), Gunnarsdóttir *et al*.^11^ (GU733718–GU733826), Delfin *et al*.^12^ (KC993902–KC994161), Ko *et al*. (2014)^13^ (KF540506-KF541055) and Chandrasekar *et al*. ^14^ (FJ383814- FJ383174). The sequences of Zheng *et al*.^7^ were retrieved from <http://www.phylotree.org/mtDNA_seqs.htm> and the sequences from Diroma *et al*.^6^ were obtained via personal communication with the authors.

**Reference**

1. Kutanan W, Kampuansai J, Srikummool M, *et al*. Complete mitochondrial genomes of Thai and Lao populations indicate an ancient origin of Austroasiatic groups and demic diffusion in the spread of Tai–Kadai languages. *Hum Genet* 2017; **136**: 85–98.

2. Zhang X, Qi X, Yang Z, *et al*. Analysis of mitochondrial genome diversity identifies new and ancient maternal lineages in Cambodian aborigines. *Nat Commun* 2013; **4**: 2599.

3. Summerer M, Horst J, Erhart G, *et al*. Large-scale mitochondrial DNA analysis in Southeast Asia reveals evolutionary effects of cultural isolation in the multi-ethnic population of Myanmar. *BMC Evol Biol* 2014; **14**: 17.

4. Li YC, Wang HW, Tian JY, *et al*. Ancient inland human dispersals from Myanmar into interior East Asia since the Late Pleistocene. *Sci Rep* 2015; **5**: 9473.

5. Peng MS, Quang HH, Dang KP, *et al*. Tracing the Austronesian footprint in mainland Southeast Asia: a perspective from mitochondrial DNA. *Mol Biol Evol* 2010; **27**: 2417–2430.

6. Diroma MA, Calabrese C, Simone D, *et al*. Extraction and annotation of human mitochondrial genomes from 1000 Genomes Whole Exome Sequencing data. *BMC Genom* 2014; **15**: S2.

7. Zheng HX, Yan S, Qin ZD, *et al*. Major population expansion of East Asians began before Neolithic time: evidence of mtDNA genomes. *PLoS ONE* 2011; **6**: e25835.

8. Zhao M, Kong QP, Wang HW, *et al*. Mitochondrial genome evidence reveals successful Late Paleolithic settlement on the Tibetan Plateau. *Proc Natl Acad Sci USA* 2009; **106**: 21230-21235.

9. Jinam TA, Hong LC, Phipps ME, *et al*. Evolutionary history of continental southeast Asians: “early train” hypothesis based on genetic analysis of mitochondrial and autosomal DNA data. *Mol Biol Evol* 2012; **29**: 3513-3527.

10. Gunnarsdóttir ED, Nandineni MR, Li M, *et al*. Larger mitochondrial DNA than Y-chromosome differences between matrilocal and patrilocal groups from Sumatra. *Nat Commun* 2011b; **2**: 228.

11. Gunnarsdottir ED, Li M, Bauchet M, Finstermeier K, Stoneking M. High-throughput sequencing of complete human mtDNA genomes from the Philippines. *Genome Res* 2011a; **21**: 1–11.

12. Delfin FS, Ko AMS, Li M, *et al*. Complete mtDNA genomes of Filipino ethnolinguistic groups: A melting pot of recent and ancient lineages in the Asia-Pacific region. *Eur J Hum Genet* 2014; **22**: 228-237.

13. Ko AMS, Chen CY, Fu Q, *et al*. Early Austronesians: into and out of Taiwan. *Am J Hum Genet* 2014; **94**: 426–436.

14. Chandrasekar A, [Kumar S](http://www.ncbi.nlm.nih.gov/pubmed/?term=Kumar%20S%5BAuthor%5D&cauthor=true&cauthor_uid=19823670), [Sreenath J](http://www.ncbi.nlm.nih.gov/pubmed/?term=Sreenath%20J%5BAuthor%5D&cauthor=true&cauthor_uid=19823670),  *et al*. Updating phylogeny of mitochondrial DNA macrohaplogroup M in India: dispersal of modern human in South Asian corridor. *PLoS ONE* 2009; **4**: e7447.

**Supplementary Table S2** Haplogroup information in all studied samples. (an excel file)

**Supplementary Table S3** Haplogroup frequencies (in percentage) observed in 22 populations. Bold letter indicates new haplogroups which were not found in our previous study of Thai/Lao populations.

|  | **MO6** | **MO7** | **KSK1** | **KSK2** | **KPW** | **KPA** | **TKH** | **LU1** | **LU2** | **LU3** | **LU4** | **YU3** | **YU4** | **YU5** | **YU6** | **CT1** | **CT2** | **CT3** | **CT4** | **CT5** | **CT6** | **CT7** |
| --- | --- | --- | --- | --- | --- | --- | --- | --- | --- | --- | --- | --- | --- | --- | --- | --- | --- | --- | --- | --- | --- | --- |
| **A+152+16362+**  **200** |  |  |  |  |  |  |  |  |  |  |  | 4 |  | 3.85 |  |  |  |  |  |  |  |  |
| A14 |  |  |  |  |  |  |  |  |  |  |  |  |  |  |  |  | 3.33 |  |  |  |  |  |
| A17 |  |  | 4 |  | 4.17 |  |  |  |  |  |  |  |  | 23.08 |  |  |  |  |  |  |  |  |
| **A5b1** |  |  |  |  |  |  |  |  |  |  |  |  |  |  |  |  |  |  |  |  | 3.45 |  |
| **B4a1a** |  |  |  |  |  |  |  |  |  |  |  |  |  |  |  |  | 3.33 |  |  |  |  |  |
| **B4a1c2** |  |  |  |  |  |  |  |  |  |  |  |  |  |  |  |  |  |  |  | 3.33 | 3.45 |  |
| B4a1c4 |  |  |  |  |  |  | 4 |  | 4.35 |  |  |  |  |  | 4 |  |  |  |  |  |  |  |
| B4a1e |  |  |  |  |  |  |  |  |  |  |  | 4 |  |  |  |  |  |  |  |  |  |  |
| B4b1a2a |  |  |  |  |  |  |  |  |  |  |  |  | 12 |  |  | 3.33 | 3.33 |  |  |  | 3.45 | 3.23 |
| **B4b1c1** |  |  |  |  |  |  |  |  |  |  |  |  |  |  |  |  |  |  | 3.33 |  |  |  |
| **B4c** |  |  |  |  |  |  |  | 4 |  |  |  |  |  |  |  |  |  |  |  |  |  |  |
| B4c1b2a |  |  |  |  |  |  |  |  | 21.74 | 4 |  |  |  |  |  |  |  |  |  |  |  |  |
| B4c2 |  |  |  |  |  |  |  | 4 |  |  |  |  |  |  |  |  | 10 |  |  |  |  |  |
| **B4c2c** |  |  |  |  |  |  | 4 | 4 |  |  |  |  |  |  |  |  |  |  |  | 3.33 |  |  |
| B4g1a |  |  |  |  |  |  | 8 |  |  |  |  |  |  |  |  |  | 3.33 | 3.33 |  |  |  | 3.23 |
| **B4g2** |  |  |  |  |  |  |  |  |  | 4 |  |  |  |  |  |  |  |  |  |  |  |  |
| **B4m** |  |  |  |  |  |  |  |  |  | 4 |  |  |  |  |  |  |  |  |  |  | 3.45 |  |
| B5a |  |  |  |  |  |  |  | 8 |  |  |  |  |  |  |  |  |  |  |  |  |  |  |
| B5a1 |  |  |  |  |  |  | 4 |  |  |  | 12.5 |  | 4 |  |  |  |  |  |  |  |  |  |
| B5a1a |  | 12.5 |  |  |  |  |  | 4 |  |  | 6.25 | 4 | 4 | 15.38 | 12 | 3.33 | 3.33 |  | 6.67 | 3.33 |  |  |
| B5a1b1 | 12.5 |  |  |  |  |  | 12 |  | 4.35 |  |  |  |  | 3.85 |  |  |  |  |  | 3.33 | 3.45 |  |
| B5a1d |  |  |  |  |  |  |  |  |  |  |  |  |  |  |  |  | 3.33 |  |  | 10 |  |  |
| **B6** |  |  |  |  |  |  |  | 4 |  |  |  |  |  |  |  |  |  |  |  |  |  |  |
| B6a |  |  |  |  |  |  |  |  |  |  |  | 12 | 4 |  |  |  |  | 3.33 | 3.33 |  |  |  |
| B6a1 |  |  |  |  |  |  |  |  |  | 4 |  |  |  |  |  |  |  |  |  |  |  |  |
| B6a1a |  |  | 44 |  | 8.33 | 20 |  |  |  |  |  | 4 |  |  |  |  |  |  |  |  | 3.45 |  |
| **C4a2b** |  |  |  |  |  |  |  |  |  |  |  |  |  |  |  |  |  | 3.33 |  |  |  |  |
| C7 |  |  |  |  |  |  |  |  |  |  |  |  |  |  |  |  |  | 3.33 |  |  |  |  |
| C7a |  |  |  |  |  |  |  |  | 4.35 |  |  | 12 | 8 |  |  |  |  |  |  |  | 17.24 | 3.23 |
| C7a1 |  |  | 24 | 7.69 | 16.67 | 4 |  |  |  |  | 18.75 |  |  |  |  |  |  |  |  |  |  |  |
| C7a2 |  |  |  |  |  |  |  |  |  | 4 |  |  |  |  | 4 |  |  |  | 3.33 |  |  |  |
| D4 |  |  |  |  |  |  |  |  |  |  |  |  |  |  |  |  |  |  |  |  | 3.45 |  |
| D4a |  |  |  |  |  |  |  |  |  |  |  | 4 |  |  | 4 |  | 3.33 |  |  |  |  |  |
| D4b2b |  |  |  |  |  |  |  |  |  |  |  |  |  |  |  |  |  |  |  |  |  | 3.23 |
| D4e1a |  |  |  |  |  |  |  |  |  |  |  |  |  |  |  |  |  |  | 3.33 |  |  |  |
| **D4e1a2** |  |  |  |  |  |  |  |  |  |  |  |  |  |  |  |  |  | 3.33 |  |  |  |  |
| D4g2a1 |  |  |  |  |  |  |  |  |  |  |  | 4 |  |  |  |  |  |  |  |  |  |  |
| D4g2a1b |  |  |  |  |  |  |  |  |  | 4 |  |  |  |  |  |  |  |  |  |  |  |  |
| D4g2a1c |  |  |  |  |  |  |  |  |  |  |  |  |  | 3.85 |  |  |  |  |  |  |  |  |
| D4h3 |  |  |  |  |  |  |  |  |  |  |  |  | 4 |  |  |  |  |  |  |  |  |  |
| D4j1a1 |  |  |  |  |  |  |  |  |  |  |  | 4 |  |  |  |  |  |  |  |  |  |  |
| **D5b4** |  |  |  |  |  |  |  |  |  |  |  | 4 |  |  |  |  |  |  | 3.33 |  |  | 6.45 |
| **F1a** |  |  |  |  |  |  |  |  | 4.35 |  |  |  |  |  |  |  |  |  |  |  |  |  |
| F1a1 |  |  |  |  |  |  |  |  |  |  |  |  | 4 |  |  |  |  |  | 6.67 |  |  |  |
| F1a1a |  |  | 8 | 46.15 | 29.17 | 4 |  |  |  |  |  |  |  |  |  | 3.33 |  | 3.33 | 3.33 |  | 3.45 | 9.68 |
| F1a1a1 |  |  |  |  |  | 12 |  |  |  | 8 |  | 4 |  | 3.85 | 4 | 6.67 | 10 |  |  | 3.33 | 6.9 |  |
| **F1a1c** |  |  |  |  |  |  |  |  |  | 4 |  |  |  |  |  |  |  |  |  |  |  |  |
| F1a1d |  |  |  |  |  |  |  |  | 4.35 |  |  |  |  |  |  |  |  |  |  |  |  |  |
| F1a2 |  |  |  |  |  |  |  |  |  |  |  |  |  |  |  |  |  |  |  |  | 3.45 |  |
| F1a3 |  |  |  |  |  |  |  |  |  |  |  |  |  |  |  |  |  | 6.67 |  |  |  |  |
| **F1a4a** |  |  |  |  |  |  |  |  |  |  |  |  |  |  |  |  |  |  |  |  | 3.45 |  |
| **F1b1+@152** |  |  |  |  |  |  |  |  |  |  |  |  |  |  |  |  |  |  | 3.33 |  |  |  |
| **F1c1a2** |  | 4.17 |  |  |  | 4 |  |  |  |  |  |  |  |  |  |  |  |  |  |  |  |  |
| F1d |  |  |  |  |  | 4 |  |  |  |  |  |  |  |  |  |  |  |  |  |  |  |  |
| F1d1 |  |  |  |  |  |  |  |  |  |  |  |  |  |  |  |  |  |  |  | 3.33 |  |  |
| **F1e** |  |  |  |  |  |  | 8 | 4 |  |  |  |  |  |  |  |  |  |  |  |  |  |  |
| F1e3 |  |  |  |  |  |  |  |  |  |  |  |  |  |  |  | 3.33 |  |  |  |  |  |  |
| F1f | 4.17 | 12.5 |  |  |  |  | 4 | 4 |  |  | 6.25 | 8 |  | 7.69 |  | 3.33 |  | 6.67 | 6.67 | 10 |  | 3.23 |
| F1g | 4.17 |  |  |  |  |  |  | 4 |  |  |  |  |  |  |  |  |  |  |  |  |  |  |
| F2 |  |  |  |  |  |  |  |  |  | 4 |  |  |  |  |  |  |  |  |  |  |  |  |
| F2b1 |  |  |  |  |  |  |  | 32 |  |  |  |  |  |  |  |  |  |  |  |  |  |  |
| **F3a** |  |  |  |  |  |  |  |  |  |  |  |  |  |  |  |  |  |  | 3.33 |  |  |  |
| F3a1 |  |  |  |  |  |  | 8 |  |  |  | 6.25 |  |  |  | 4 | 3.33 |  |  |  |  |  |  |
| **F3b** |  |  |  |  |  |  |  |  |  |  |  |  |  |  |  |  |  |  | 3.33 |  |  |  |
| **F3b (152)** |  |  |  |  |  |  |  |  |  | 8 |  |  |  |  |  |  |  |  |  |  |  |  |
| F4a2 |  |  |  |  |  |  | 4 |  |  |  |  |  |  |  |  |  |  |  |  |  |  | 3.23 |
| **G2** |  |  |  |  |  |  |  |  |  |  |  |  |  |  | 12 |  |  |  |  |  |  |  |
| **G2a1** |  | 8.33 |  |  |  |  | 4 |  |  |  |  |  |  | 7.69 |  |  |  |  |  |  | 10.34 |  |
| **G2a1d2** |  |  |  |  |  |  |  |  |  |  |  |  |  |  |  |  | 3.33 | 3.33 |  |  |  |  |
| G2a1d2a |  |  |  |  |  |  |  |  |  |  |  |  |  | 3.85 |  |  |  |  |  |  |  |  |
| G2b1a |  |  |  |  |  |  | 16 |  |  | 4 |  |  |  |  |  |  |  |  |  |  |  |  |
| G2b1a1 |  |  | 4 | 7.69 | 4.17 |  |  |  |  |  |  |  |  |  |  |  |  |  |  |  |  |  |
| G3b2 |  |  |  |  |  |  |  |  |  |  |  |  |  |  | 4 |  |  |  |  |  |  |  |
| **H13a2a** |  |  |  |  |  |  |  |  |  |  |  |  |  |  |  | 3.33 |  |  |  |  | 3.45 |  |
| **I1b** |  | 4.17 |  |  |  |  |  |  |  |  |  |  |  |  |  |  |  |  |  |  |  |  |
| M | 25 |  |  |  |  | 12 |  |  |  |  |  | 4 |  |  |  |  |  |  |  | 3.33 |  |  |
| **M10a1** |  | 4.17 |  |  |  |  |  |  |  |  |  |  |  |  |  |  |  |  |  |  |  |  |
| M1'20'51 |  | 4.17 |  |  |  |  |  |  |  |  |  |  |  |  |  |  |  |  |  |  |  |  |
| M12a1a | 25 |  |  |  |  |  |  |  |  | 4 |  |  | 8 |  |  |  | 3.33 |  |  |  |  | 3.23 |
| **M12a2** |  |  |  |  |  |  |  |  |  |  |  |  |  |  |  |  | 3.33 | 3.33 |  | 6.67 |  |  |
| M12b1a2 |  |  |  |  |  |  |  |  |  |  |  |  |  |  |  |  | 3.33 |  |  |  |  |  |
| M12b1b |  |  |  |  |  |  |  |  |  |  |  |  | 4 |  |  | 3.33 |  |  |  |  |  |  |
| **M12b2** |  |  |  |  |  |  |  |  |  |  |  |  |  |  |  |  |  |  |  |  | 3.45 |  |
| **M13a2** |  |  |  |  |  | 4 |  |  |  |  |  |  |  |  |  |  |  |  |  |  |  |  |
| **M13b1** |  |  |  |  |  |  |  |  |  |  |  |  |  |  |  |  |  |  |  |  |  | 3.23 |
| M13c |  | 12.5 |  |  |  |  |  |  |  |  |  |  |  |  |  |  |  |  |  |  |  |  |
| M17a |  |  |  |  |  |  |  |  |  |  |  |  |  |  |  |  |  |  |  | 3.33 |  |  |
| M17c |  |  |  |  |  |  |  |  |  |  |  |  |  |  |  | 3.33 | 3.33 | 10 | 3.33 |  |  |  |
| **M17c1a1** |  |  |  |  |  |  |  |  |  |  |  |  |  |  |  | 6.67 | 3.33 |  |  |  |  |  |
| **M17c1a1a** |  |  |  |  |  |  |  |  |  |  |  |  |  |  |  | 3.33 |  |  |  |  |  |  |
| M20 |  |  |  |  | 4.17 |  |  |  |  |  |  |  |  |  |  |  |  |  |  |  |  |  |
| **M21a** |  | 4.17 |  | 7.69 | 8.33 | 12 |  |  |  |  |  |  |  |  |  |  |  |  |  |  |  |  |
| M21b |  |  |  |  |  |  |  |  |  |  |  |  |  |  |  |  |  |  |  | 6.67 |  |  |
| **M21b2** |  |  |  |  |  |  |  |  |  |  |  |  |  |  |  |  | 3.33 | 3.33 |  |  |  | 3.23 |
| M24a |  |  |  |  |  |  |  |  |  |  |  |  |  |  |  |  |  | 3.33 |  |  |  |  |
| M24b |  | 4.17 |  |  |  |  |  |  |  |  |  |  |  |  |  |  |  |  |  |  |  |  |
| **M2a1a** |  |  |  |  |  |  |  |  |  |  |  |  |  |  |  |  |  | 3.33 |  |  |  |  |
| **M30** |  | 4.17 |  |  |  |  |  |  |  |  |  |  |  |  |  |  |  |  |  |  |  |  |
| **M32'56** |  |  |  |  |  |  |  |  |  |  |  |  |  |  |  |  |  |  |  |  |  | 3.23 |
| **M37e2** |  |  |  |  |  |  |  |  |  |  |  |  |  |  |  |  |  |  |  |  |  | 3.23 |
| **M40a1** | 4.17 |  |  |  |  |  |  |  |  |  |  |  |  |  |  |  |  |  |  |  |  |  |
| M45a |  | 4.17 |  |  |  |  |  |  |  |  |  |  |  |  |  |  |  |  |  |  |  |  |
| **M50a1** |  |  |  |  |  |  |  |  |  |  |  |  |  |  |  |  |  |  | 3.33 |  |  |  |
| M51a |  |  |  |  |  |  |  |  |  |  |  |  |  |  |  |  |  |  | 3.33 |  |  |  |
| **M51a1a** |  |  |  |  |  |  |  |  |  |  |  |  |  |  |  |  | 3.33 |  |  |  |  |  |
| **M55** |  |  |  |  |  |  |  |  |  |  |  |  | 8 |  |  |  |  |  |  |  |  |  |
| M5b |  |  |  |  |  |  |  |  |  |  |  |  | 4 |  |  |  |  |  |  |  |  |  |
| M5c1 |  |  |  |  |  |  |  |  |  |  |  |  |  |  |  |  |  |  |  |  |  | 3.23 |
| M61 |  |  |  |  |  |  |  |  |  |  |  | 4 |  |  |  |  |  |  |  |  |  |  |
| M6a1a |  | 4.17 |  |  |  |  |  |  |  |  |  |  |  |  |  |  |  |  |  |  |  |  |
| M71 (151T) |  |  |  |  |  | 8 |  |  |  |  |  |  |  |  |  |  |  |  |  |  |  |  |
| M71a |  |  |  |  |  |  |  |  |  |  |  |  |  |  |  |  |  |  | 6.67 |  |  |  |
| M71a2 |  |  |  |  |  |  | 4 |  |  |  |  | 4 |  |  |  |  |  |  |  | 6.67 |  | 6.45 |
| M71b |  |  |  |  |  |  | 4 |  |  |  |  |  | 4 |  |  |  | 3.33 |  |  |  |  |  |
| **M71c** |  |  |  |  |  |  |  |  |  |  |  |  |  |  |  |  |  |  |  |  |  | 3.23 |
| M72a |  |  |  |  |  |  |  |  |  |  |  |  |  |  |  |  |  | 3.33 |  |  |  |  |
| M73 |  |  |  |  |  |  |  |  |  |  |  |  |  |  |  |  |  |  | 3.33 |  |  |  |
| **M73a1** |  |  |  |  |  |  |  |  |  |  |  |  |  |  |  | 3.33 | 3.33 |  |  |  |  |  |
| **M73b** |  |  |  |  |  |  |  |  |  |  |  |  |  |  |  |  |  |  |  |  | 3.45 |  |
| M74 |  |  |  |  |  |  |  |  |  |  |  |  |  |  |  |  |  | 3.33 |  |  |  |  |
| M74a |  |  |  |  |  |  |  | 4 |  |  |  |  |  |  |  | 3.33 |  |  |  |  |  |  |
| M75 |  |  |  |  |  |  |  |  |  |  |  |  | 4 |  |  |  |  |  |  |  |  | 3.23 |
| M76 |  |  |  |  |  |  |  |  |  |  |  |  |  |  |  |  |  | 3.33 | 3.33 | 3.33 |  |  |
| M76a |  |  |  |  |  |  |  |  |  | 8 |  |  |  |  |  |  |  |  |  |  |  |  |
| M79 |  |  |  |  |  |  |  |  |  |  |  |  |  |  |  |  | 3.33 |  |  |  |  |  |
| **M7** |  |  |  |  |  |  |  |  |  |  |  |  |  |  |  |  |  |  | 3.33 |  |  |  |
| **M7b** |  |  |  |  |  |  |  |  |  |  |  |  |  |  |  |  |  |  |  |  |  | 6.45 |
| M7b1a1 |  |  |  |  | 4.17 |  |  |  |  |  | 6.25 |  |  | 7.69 |  | 3.33 |  | 3.33 |  | 3.33 |  |  |
| M7b1a1 (16192T) | 4.17 |  |  |  |  |  | 4 |  |  |  | 12.5 |  |  |  | 8 |  |  | 3.33 |  | 3.33 |  | 3.23 |
| M7b1a1a |  |  |  |  |  |  |  |  |  |  | 6.25 |  |  |  |  |  |  | 10 |  |  |  |  |
| M7b1a1a2 |  |  |  |  |  |  |  |  |  |  |  |  |  | 3.85 |  |  |  |  |  |  |  |  |
| M7b1a1a3 |  |  |  |  |  |  |  | 4 | 8.7 | 4 | 6.25 |  | 4 |  |  | 10 |  | 3.33 | 3.33 |  |  |  |
| M7b1a1b | 4.17 |  |  |  |  |  |  | 12 |  | 8 |  | 8 |  |  |  |  |  |  |  |  |  |  |
| M7b1a1d1 |  |  |  |  |  |  | 4 |  |  | 4 |  |  |  |  |  |  |  |  |  | 6.67 |  |  |
| M7b1a1e |  |  |  |  |  |  |  |  |  | 4 |  |  |  |  |  |  |  |  |  |  |  |  |
| M7b1a1e1 | 4.17 |  |  |  |  |  |  |  | 21.74 |  |  |  |  |  |  |  |  |  | 3.33 |  |  |  |
| M7b1a1f |  | 4.17 |  |  | 4.17 |  |  |  |  | 4 |  |  | 8 |  |  |  |  |  |  |  |  |  |
| **M7b1a1g** |  |  |  |  |  |  |  |  |  |  |  |  |  |  |  |  |  | 3.33 |  |  |  |  |
| **M7b1a1h** |  |  |  |  |  |  |  |  |  |  | 18.75 |  |  |  |  |  |  |  |  |  |  |  |
| M7c1a |  |  |  |  |  |  |  |  |  |  |  |  |  |  |  | 3.33 |  |  |  |  |  | 9.68 |
| M7c1c2 |  |  |  |  |  |  |  |  |  |  |  |  | 4 | 3.85 |  | 3.33 |  |  |  |  |  |  |
| **M7c1c3** |  |  |  |  |  |  |  |  |  |  |  |  |  |  |  | 6.67 |  |  |  |  |  |  |
| M7c2a |  |  |  |  |  |  |  |  |  |  |  |  |  |  |  |  | 3.33 |  |  |  | 3.45 |  |
| **M7c2b** |  |  |  |  |  |  |  |  |  | 4 |  |  |  |  |  |  |  |  |  |  |  |  |
| M8a2a1 |  |  |  |  |  |  |  |  |  |  |  |  |  |  | 28 |  |  |  |  |  |  |  |
| M91a |  | 4.17 |  |  |  | 12 |  |  |  |  |  |  |  |  |  |  |  | 6.67 |  |  |  |  |
| M9a1b1 | 4.17 |  |  |  |  |  |  |  |  |  |  |  |  |  |  |  |  |  |  |  |  |  |
| M9a5 |  |  |  |  |  |  |  |  |  |  |  |  |  |  |  |  | 3.33 |  |  |  |  |  |
| N10a |  |  |  |  |  |  |  |  |  |  |  |  |  |  |  | 13.33 |  |  |  |  |  |  |
| N21 (195C) |  |  |  |  |  |  |  |  |  |  |  | 4 |  |  |  |  | 3.33 |  |  |  |  | 3.23 |
| N21a |  |  |  |  |  | 4 |  |  |  |  |  |  |  |  |  |  |  |  |  |  |  |  |
| N22 |  |  |  |  |  |  |  |  |  |  |  |  | 4 |  |  |  |  |  |  |  |  |  |
| N8 |  |  |  | 30.77 |  |  |  |  |  |  |  |  |  |  |  |  |  |  |  |  |  |  |
| N9a |  |  |  |  |  |  |  | 8 |  |  |  |  |  |  | 8 |  |  |  |  |  |  |  |
| N9a10 |  |  |  |  |  |  |  |  |  |  |  |  |  |  |  |  |  |  |  | 3.33 | 3.45 | 3.23 |
| N9a6 |  |  |  |  |  |  |  |  |  |  |  | 4 |  |  |  |  |  |  |  |  |  | 3.23 |
| **R11'B6** |  |  |  |  |  |  |  |  |  |  |  |  |  |  |  |  |  |  | 3.33 |  |  |  |
| **R2+13500** |  |  |  |  |  |  | 4 |  |  |  |  |  |  | 3.85 |  |  |  |  |  |  |  |  |
| **R21** |  |  |  |  |  |  |  |  |  |  |  |  |  |  |  |  | 3.33 |  |  |  |  |  |
| R22 |  | 4.17 |  |  |  |  |  |  |  |  |  |  |  |  |  |  |  |  | 3.33 |  | 3.45 |  |
| **R23** |  |  |  |  |  |  |  |  |  |  |  |  |  |  |  |  |  |  |  | 3.33 |  |  |
| R9b1 |  | 4.17 |  |  |  |  |  |  |  |  |  |  |  |  |  |  |  |  | 3.33 |  |  |  |
| R9b1a1a |  |  | 16 |  | 16.67 |  |  |  |  | 4 |  |  |  |  |  | 3.33 |  |  |  | 3.33 |  |  |
| R9b1a2 |  |  |  |  |  |  |  |  |  |  |  |  |  |  |  |  |  |  | 3.33 |  |  |  |
| **R9b1a2b** |  |  |  |  |  |  | 4 |  |  |  |  |  |  |  |  |  |  |  |  |  |  |  |
| R9b1a3 | 4.17 |  |  |  |  |  |  |  | 21.74 |  |  | 4 | 4 | 3.85 | 4 |  |  |  |  |  | 3.45 |  |
| R9b1b | 4.17 |  |  |  |  |  |  |  |  |  |  |  |  |  | 4 |  |  |  |  |  |  |  |
| R9b2 |  |  |  |  |  |  |  |  | 4.35 |  |  |  | 4 |  |  | 3.33 |  |  |  | 3.33 |  | 3.23 |
| R9c1b1 |  |  |  |  |  |  |  |  |  | 4 |  |  |  | 3.85 |  |  |  |  |  |  |  |  |
| **U1a1c1a** |  |  |  |  |  |  |  |  |  |  |  |  |  |  |  |  |  |  |  | 3.33 |  |  |
| **U1a1c1d** |  |  |  |  |  |  |  |  |  |  |  |  |  |  |  |  | 3.33 |  | 3.33 |  |  |  |
| **U2a1b** |  |  |  |  |  |  |  |  |  |  |  |  |  |  |  |  |  |  |  |  | 3.45 |  |
| **U2a2** |  |  |  |  |  |  |  |  |  |  |  |  |  |  |  |  | 3.33 |  |  |  |  |  |
| W3a1b |  |  |  |  |  |  |  |  |  |  |  |  |  |  |  |  |  |  |  |  | 3.45 |  |

**Supplementary Table S4** Random forests confusion matrix and classification error for the ABC analysis of Central Thai origins.

|  | **Demic diffusion** | **Cultural diffusion** | **Continuous migration** | **Classification error** |
| --- | --- | --- | --- | --- |
| **Demic diffusion** | 7257 | 214 | 2529 | 0.27 |
| **Cultural diffusion** | 254 | 9288 | 458 | 0.07 |
| **Continuous migration** | 4283 | 721 | 4996 | 0.5 |

**Supplementary Table S5** Votes assigned to each model by the Random Forest procedure and posterior probability for the selected model in the ABC analysis of Central Thai origins.

| **selected model** | **votes demic diffusion model** | **votes cultural diffusion model** | **votes continuous migration model** | **Posterior Probability** |
| --- | --- | --- | --- | --- |
| **1** | 0.515 | 0.081 | 0.404 | 0.604 |

**Supplementary Table S6** Parameters estimation for the demic diffusion and continuous migration model in the ABC analysis of Central Thai origins. NcAA is the current effective population size of Austroasiatic populations, NcCT is the current central Thai effective population size, NcDAI is the current effective population size for Southern Chinese populations, M1 is the migration rate to Austroasiatic groups from Central Thai populations and M2 is the migration rate to Central Thai populations from Austroasiatic groups.

| **Demic diffusion** | | | | | |
| --- | --- | --- | --- | --- | --- |
|  | **Median** | **Mode** | **95% HPD-LowB** | **95% HPD-UppB** | **R.Squared** |
| **NcAA** | 13,617 | 13,861 | 9,485 | 17,960 | 0.925 |
| **NcCT** | 211,902 | 129,884 | 31,972 | 814,367 | 0.773 |
| **NcDAI** | 31,523 | 20,851 | 6,713 | 84,128 | 0.788 |
| **Continous migration** | | | | | |
|  | **Median** | **Mode** | **95% HPD-LowB** | **95% HPD-UppB** | **R.Squared** |
| **M1 (CT->AA)** | 0.01 | 0.01 | 0.01 | 0.171 | 0.078 |
| **M2 (AA->CT)** | 0.01 | 0.01 | 0.01 | 0.142 | 0.195 |
| **NcAA** | 14,052 | 14,109 | 9,257 | 18,822 | 0.924 |
| **NcCT** | 137,565 | 74,134 | 9,392 | 718,654 | 0.737 |
| **NcDAI** | 27,446 | 17,297 | 3,990 | 77,257 | 0.755 |

**Supplementary Table S7** Random forests confusion matrix and classification error for the ABC analysis of the relationships between populations from different MSEA language families.

|  | **Model 1** | **Model 2** | **Model 3** | **Model 4** | **Model 5** | **Classification error** |
| --- | --- | --- | --- | --- | --- | --- |
| **Model 1** | 8154 | 1217 | 207 | 251 | 171 | 0.18 |
| **Model 2** | 1624 | 7789 | 481 | 47 | 59 | 0.22 |
| **Model 3** | 193 | 380 | 9035 | 83 | 309 | 0.1 |
| **Model 4** | 171 | 86 | 84 | 8735 | 924 | 0.13 |
| **Model 5** | 258 | 122 | 341 | 937 | 8342 | 0.17 |

**Supplementary Table S8** Votes assigned to each model by the Random Forest procedure and posterior probability for the selected model in the ABC analysis of the relationships between populations from different MSEA language families.

| **Selected model** | **Votes model 1** | **Votes model 2** | **Votes model 3** | **Votes model 4** | **Votes model 5** | **Post. Prob.** |
| --- | --- | --- | --- | --- | --- | --- |
| 1 | 0.509 | 0.311 | 0.037 | 0.112 | 0.031 | 0.656 |
